# HLA Class I and II Variants as Potential Determinants of Clinical Severity and Mortality in Patients with COVID-19: A Prospective Study from Saudi Arabia

**DOI:** 10.3390/biomedicines14061220

**Published:** 2026-05-28

**Authors:** Jawaher A. Alsubait, Dalal S. Alshaya, Fatimah F. Alghnnam, Mashael J. Abu-Alola, Marie Fe F. Bohol, Saltana A. Alhowaiti, Abdullah Al Marzan, Arwa A. Al-Qahtani, Esra’a Abudouleh, Tarek Owaidah, Fatimah Alhamlan, Ahmed Al-Qahtani

**Affiliations:** 1 Department of Biology, College of Science, Princess Nourah Bint Abdulrahman University, P.O. Box 84428, Riyadh 11671, Saudi Arabia; jwahrbdallh978@gmail.com (J.A.A.); dsalshaya@pnu.edu.sa (D.S.A.); 2 Department of Infection and Immunity, Research Centre, King Faisal Specialist Hospital & Research Centre, Riyadh 11211, Saudi Arabia; falghnnam@kfshrc.edu.sa (F.F.A.); malsaeedi@kfshrc.edu.sa (M.J.A.-A.); mbohol@kfshrc.edu.sa (M.F.F.B.); salhuwaiti@kfshrc.edu.sa (S.A.A.); falhamlan@kfshrc.edu.sa (F.A.); 3 Department of Biochemistry and Molecular Biology, Shahjalal University of Science and Technology, Sylhet 3114, Bangladesh; marzansust16@gmail.com; 4 Department of Data Science, Toxicology Society of Bangladesh, Dhaka 1000, Bangladesh; 5 Department of Family Medicine, College of Medicine, Imam Mohammad Ibn Saud Islamic University (IMSIU), Riyadh 11432, Saudi Arabia; arahalqahtani@imamu.edu.sa; 6 Department of Botany and Microbiology, College of Science, King Saud University, Riyadh 11451, Saudi Arabia; eabudouleh@ksu.edu.sa; 7 Department of Pathology and Laboratory Medicine, King Faisal Specialist Hospital & Research Centre, Riyadh 11211, Saudi Arabia; towaidah@kfshrc.edu.sa; 8 Department of Microbiology and Immunology, College of Medicine, Alfaisal University, Riyadh 11533, Saudi Arabia

**Keywords:** COVID-19, SARS-CoV-2, human leukocyte antigen, genetic polymorphisms, disease severity, Saudi Arabia

## Abstract

**Background/Objectives:** Genetic variation in human leukocyte antigen (HLA) genes may contribute to inter-individual differences in infectious-disease susceptibility and clinical outcomes. This study aimed to determine the genotype frequency of HLA Class I and Class II loci in patients with COVID-19 in Saudi Arabia and to examine their associations with survival and clinical severity. **Methods:** A prospective observational study was conducted at King Faisal Specialist Hospital and Research Centre (KFSH&RC), Riyadh, Saudi Arabia, from January 2022 to December 2023. Genomic DNA was extracted, and polymerase chain reaction-sequence specific oligonucleotide (PCR-SSO) testing was performed to screen HLA genetic variation. Patients were grouped by survival status (recovered or deceased) and clinical severity: Stage A (asymptomatic), Stage B (mild), Stage C (moderate), and Stage D (severe). **Results:** In total, 123 patients with COVID-19 were included; 102 (82.9%) recovered and 21 (17.1%) died. ICU admission was more frequent among deceased patients than among recovered patients (95.2% versus 51.0%, *p* = 0.0001). At the locus level, HLA-DPB1 represented the largest proportion of HLA calls (21%). In call-position-specific allele-group analyses, B*15 in Allele 1 (14.3% versus 1.0%, *p* = 0.016), C*06 in Allele 2 (42.9% versus 18.6%, *p* = 0.023), DRB1*10 in Allele 1 (19.0% versus 4.9%, *p* = 0.045), and DQB1*05 in Allele 1 (33.3% versus 11.8%, *p* = 0.021) were significantly more frequent among deceased patients, whereas DQB1*03 in Allele 1 was significantly more frequent among recovered patients (45.1% versus 14.3%, *p* = 0.013). Severity analyses showed call-position-specific differences involving C*15, C*06, B*14, B*39, B*53, and DQB1*03. Vaccination status did not differ significantly by survival status or across the four clinical severity stages. **Conclusions:** Selected HLA Class I and Class II allele groups may be associated with COVID-19 survival and clinical severity patterns in this Saudi cohort. These findings should be interpreted cautiously given the cohort size and call-position-specific nature of the analyses.

## 1. Introduction

Coronavirus disease 2019 (COVID-19), caused by SARS-CoV-2, created a major global health-care and economic crisis beginning in 2020 [1,2]. Although the acute pandemic phase has subsided, COVID-19 continues to occur and remains clinically relevant, particularly among individuals at increased risk of severe disease. Clinical outcomes vary substantially among infected individuals. In addition to established risk factors such as older age, male sex, and comorbidities [3], host genetic variation may contribute to differences in COVID-19 severity and mortality [4,5,6].

The human leukocyte antigen (HLA) system comprises a cluster of highly polymorphic genes located on chromosome 6 that encode major histocompatibility complex (MHC) molecules. These molecules are expressed on cell surfaces and present pathogen-derived peptides to T lymphocytes, thereby initiating and shaping adaptive immune responses [7]. Because HLA molecules are central to antigen presentation, variation in HLA genes may influence host susceptibility, resistance, and clinical outcomes across infectious diseases. HLA alleles are highly polymorphic, and populations with different HLA profiles may generate distinct immune responses against pathogenic microorganisms [8,9]. HLA polymorphisms have been implicated in susceptibility or resistance to infectious diseases including dengue [10], influenza [11,12], malaria [13], Middle East respiratory syndrome coronavirus (MERS-CoV) [14], severe acute respiratory syndrome coronavirus (SARS-CoV) [15], and tuberculosis [16].

Several studies have investigated associations between HLA polymorphisms and COVID-19 severity [5,6,17]. In patients from Romania and Moldova, Vică et al. reported that HLA-B*27 and HLA-B*50 were associated with increased risk of severe COVID-19, whereas HLA-A*33 and HLA-C*15 showed protective associations; HLA-A*03 and HLA-DQB1*02 were also suggested to confer protection against severe disease [6]. A study by Hakimi et al. [17] revealed that individuals carrying HLA-A*23, HLA-DRB1*10, and HLA-DRB1*13 had higher odds of developing severe COVID-19. A substantial decline in lymphocyte count and an increased likelihood of thrombosis were also observed among COVID-19 patients carrying HLA-A*23 [17]. In the Saudi population, Hajeer et al. performed next-generation sequencing and reported that HLA-B*51:01:01G was associated with lower risk of severe COVID-19, whereas HLA-B*50:01:01G, HLA-C*06:02:01G, and HLA-DRB1*07:01:01G were associated with increased predisposition to disease severity [5]. Additional recent studies from Spain, Vietnam, and Iran have further supported the population-specific relationship between HLA polymorphisms and COVID-19 susceptibility, severity, or mortality [17,18,19]. Therefore, HLA genetic diversity may partly explain inter-individual and population-level differences in the COVID-19 clinical course. With this background, we aimed to determine HLA Class I and Class II genotype distributions using polymerase chain reaction-sequence specific oligonucleotide (PCR-SSO) testing in patients with COVID-19 in Saudi Arabia and to examine their associations with survival and clinical severity.

## 2. Materials and Methods

### 2.1. Ethical Considerations

The proposed research study underwent careful review, and ethical approval was obtained from the Ethics Review Committee (ERC) at King Faisal Specialist Hospital and Research Centre (KFSH&RC), Riyadh, Saudi Arabia (Reference Number: 2201086). This study was conducted in accordance with the principles of the 1975 Declaration of Helsinki. Written informed consent was obtained from participants prior to enrollment. The identity of the research participants was kept strictly confidential by removing their identifiers from the patient data and biological samples. Patient data were stored electronically on a password-protected desktop computer, and access was limited to the principal investigator and members of the research team.

### 2.2. Study Design, Setting, and Patients

A prospective observational study was conducted at King Faisal Specialist Hospital and Research Centre (KFSH&RC), Riyadh, Saudi Arabia, from January 2022 to December 2023. The study included 123 laboratory-confirmed COVID-19 patients admitted to the general ward or intensive care unit (ICU). COVID-19 was confirmed by detection of SARS-CoV-2 nucleic acid from nasopharyngeal, oral, or tracheal swabs using real-time reverse transcriptase-polymerase chain reaction (RT-PCR). Patients were categorized according to survival outcome as recovered or deceased. They were further classified according to clinical severity into Stage A (asymptomatic infection), Stage B (mild infection), Stage C (moderate infection), and Stage D (severe infection).

### 2.3. Demographic and Clinical Data

Before commencing data collection, written informed consent was obtained from the patients or their guardians for enrollment. Demographic and clinical data were collected from all 123 COVID-19 patients. Data were entered into REDCap, a secure electronic data-capture platform hosted at KFSH&RC, Riyadh, Saudi Arabia. The following clinically relevant demographic and clinical variables were recorded: age, sex, body mass index, vaccination status, creatinine, coagulation/hemostatic markers (incidence of thrombosis, platelet, international normalized ratio [INR], prothrombin time [PT], partial thromboplastin time [PTT], D-dimer, and fibrinogen), ICU admission, and length of hospital stay.

### 2.4. HLA Genotyping

Genomic DNA was extracted from blood samples using the DNeasy commercial kit (Qiagen, Valencia, CA, USA) according to the manufacturer’s protocol. HLA genotyping was performed using polymerase chain reaction-sequence specific oligonucleotide (PCR-SSO) testing with the LIFECODES^®^ HLA SSO Typing kit (Immucor Inc., Norcross, GA, USA). To achieve optimal HLA genotype accuracy on the FLEXMAP 3D^®^ system (Luminex Corporation, Austin, TX, USA), the RSSOW1A, RSSOW1B, and RSSOW1C kits from One Lambda, Inc. (Thermo Fisher Scientific, West Hills, CA, USA) were used. Genotypes were determined for HLA-A, HLA-B, HLA-C, HLA-DRB1, HLA-DRB3, HLA-DRB4, HLA-DRB5, HLA-DQA1, HLA-DQB1, HLA-DPA1, HLA-DPB1, HLA-DOA, HLA-DOB, HLA-DMA, and HLA-DMB. For class-based interpretation, HLA-A, HLA-B, and HLA-C were categorized as HLA Class I loci, whereas HLA-DRB1, HLA-DRB3, HLA-DRB4, HLA-DRB5, HLA-DQA1, HLA-DQB1, HLA-DPA1, HLA-DPB1, HLA-DOA, HLA-DOB, HLA-DMA, and HLA-DMB were categorized as HLA Class II loci. For graphical and descriptive analyses, the two allele calls generated for each locus were reported as Allele 1 and Allele 2 call positions; these labels indicate genotyping call order only and should not be interpreted as parental origin or phased inheritance.

### 2.5. Statistical Analysis

Data were entered, processed, and analyzed using the Statistical Package for the Social Sciences (SPSS) for Windows, version 24.0 (IBM Corporation, Armonk, NY, USA). Numerical variables were expressed as mean ± standard deviation (SD) or median with interquartile range (IQR), as appropriate. Categorical variables were compared using Pearson’s chi-square (χ^2^) test or Fisher’s exact test, as appropriate. The independent samples *t*-test or Mann–Whitney U test was applied for continuous variables according to data distribution. Vaccination status was compared between survival groups using Fisher’s exact test and across the four clinical severity stages using Pearson’s chi-square test. Because several HLA allele-group comparisons contained sparse or zero-cell counts, Fisher’s exact test was used for allele-group significance testing. Exploratory interaction analyses were performed using logistic regression models to test age × sex, BMI × sex, and age × BMI interaction terms for mortality and clinical severity outcomes. Age was modeled per 10-year increase, BMI per 5 kg/m^2^ increase, and sex as male versus female. Interaction effects were reported as odds ratios (ORs) with 95% confidence intervals (CIs). A *p* value of <0.05 was considered statistically significant.

## 3. Results

### 3.1. Demographic and Clinical Characteristics of Study Patients

The study included 123 Saudi patients with laboratory-confirmed COVID-19. Patients were categorized according to survival outcome into recovered (*n* = 102) and deceased (*n* = 21) groups. Demographic and clinical characteristics are summarized in Table 1. No statistically significant differences were observed between the two groups with respect to sex distribution, age, body mass index, coagulation/hemostatic markers, renal markers, or length of hospital stay (*p* ≥ 0.05). However, ICU admission was significantly more frequent among deceased patients than among recovered patients (95.24% versus 50.98%, *p* = 0.0001).

Patients were further assessed according to COVID-19 clinical severity stage: Stage A (asymptomatic infection), Stage B (mild infection), Stage C (moderate infection), and Stage D (severe infection). Patients with Stage C+D disease had a significantly higher median age than those with Stage A+B disease (*p* = 0.001). Median platelet count was lower in the Stage C+D group (*p* = 0.022), whereas fibrinogen level was higher in the Stage C+D group than in the Stage A+B group (*p* = 0.004). In the Stage A+B+C versus Stage D comparison, ICU admission was significantly more frequent in Stage D patients (*p* < 0.0001), and mean prothrombin time was lower in Stage D patients (*p* = 0.009) (Table 2).

### 3.2. Distribution of HLA Locus Calls

At the locus level, HLA-DPB1 represented the largest proportion of HLA calls in the cohort (21%), followed by HLA-A (16%), HLA-B (16%), and HLA-DRB1 (12%). HLA-C accounted for 10% of calls, whereas HLA-DQA1, HLA-DQB1, HLA-DOB, HLA-DPA1, HLA-DRB3, and HLA-DRB5 represented 5%, 4%, 4%, 3%, 3%, and 2%, respectively. HLA-DRB4, HLA-DMA, HLA-DMB, and HLA-DOA were the least represented loci, each accounting for approximately 1% of calls.

### 3.3. HLA Allele-Group Distribution Across Allele 1 and Allele 2 Calls

**Figure 1:**
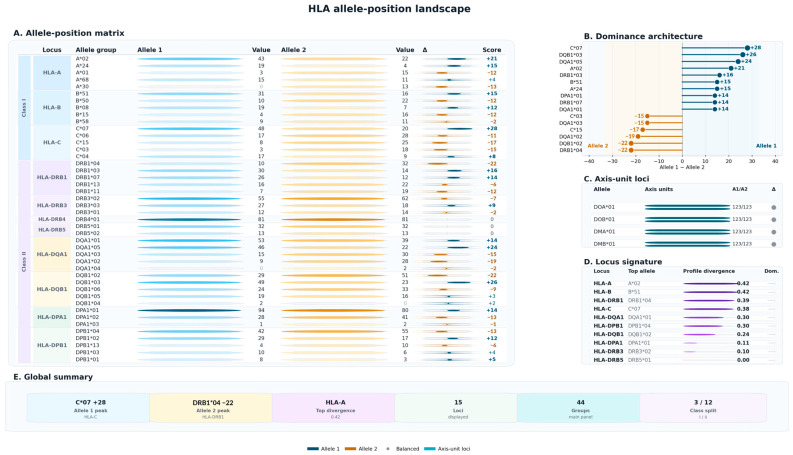
HLA allele-position landscape across HLA loci. (**A**) Allele-position matrix showing allele-group values for Allele 1 and Allele 2 call positions across Class I and Class II HLA loci. Rows are grouped by locus and ordered by the maximum observed value. (**B**) Dominance architecture showing the direction and magnitude of Allele 1–Allele 2 separation for the leading allele groups. (**C**) Axis-unit loci showing balanced call-position values for DOA*01, DOB*01, DMA*01, and DMB*01. (**D**) Locus signature summarizing the top allele group and profile divergence for each locus. (**E**) Global summary of the allele-position landscape, including Allele 1 and Allele 2 peaks, top-divergence locus, number of loci displayed, number of allele groups in the main panel, and Class I/Class II split. Allele 1 and Allele 2 represent genotyping call positions and do not imply parental origin or phased inheritance.

HLA allele-group distributions were compared across the two allele-call positions, designated Allele 1 and Allele 2, for each HLA locus (Figure 1A–E). These allele-call positions reflect the two HLA calls generated during genotyping and do not imply parental origin or phased inheritance.

In the allele-position matrix, the leading HLA-A allele groups showed a predominance of A*02 in Allele 1 compared with Allele 2 (43 versus 22; Δ = +21), followed by A*24 (19 versus 4; Δ = +15). In contrast, A*01 and A*30 were higher in Allele 2 than in Allele 1 (3 versus 15 and 0 versus 13, respectively). For HLA-B, B*51 showed the strongest Allele 1 predominance (31 versus 16; Δ = +15), while B*50 and B*15 were higher in Allele 2 (10 versus 22 and 4 versus 16, respectively). Among HLA-C allele groups, C*07 showed the strongest Allele 1 predominance in the entire matrix (48 versus 20; Δ = +28), whereas C*15, C*03, and C*06 were higher in Allele 2.

For HLA-DRB1, DRB1*03 and DRB1*07 were higher in Allele 1 (30 versus 14 and 26 versus 12, respectively), whereas DRB1*04 showed the strongest Allele 2 predominance (10 versus 32; Δ = −22), followed by DRB1*11 (7 versus 19; Δ = −12). HLA-DRB3 showed moderate Allele 2 predominance for DRB3*02 (55 versus 62) and Allele 1 predominance for DRB3*03 (27 versus 18). HLA-DRB4*01, DRB5*01, and DRB5*02 were balanced across Allele 1 and Allele 2 call positions.

For HLA-DQ and HLA-DP loci, DQA1*05 and DQA1*01 were higher in Allele 1, whereas DQA1*02 and DQA1*03 were higher in Allele 2. DQB1*03 showed marked Allele 1 predominance (49 versus 23; Δ = +26), whereas DQB1*02 showed marked Allele 2 predominance (29 versus 51; Δ = −22). DPA1*01 was higher in Allele 1 (94 versus 80), while DPA1*02 was higher in Allele 2 (28 versus 41). For HLA-DPB1, DPB1*04 was higher in Allele 2 (42 versus 55), whereas DPB1*02 was higher in Allele 1 (29 versus 17).

The dominance architecture panel highlighted C*07 as the strongest Allele 1-enriched allele group (Δ = +28) and DRB1*04 and DQB1*02 as the strongest Allele 2-enriched allele groups (both Δ = −22). The locus signature panel showed the highest profile divergence for HLA-A and HLA-B, followed by HLA-DRB1 and HLA-C. The axis-unit loci, including DOA*01, DOB*01, DMA*01, and DMB*01, were balanced across both call positions, each showing 123/123 axis-unit values. Extended supporting visualizations for the allele-group analysis are provided in Supplementary Figures S1–S5, including the complete percentage rows, paired call-position profiles, full dominance architecture, locus-signature details, and global summary ribbon.

### 3.4. Two-Field HLA Allele Distribution Across Allele 1 and Allele 2 Calls

**Figure 2:**
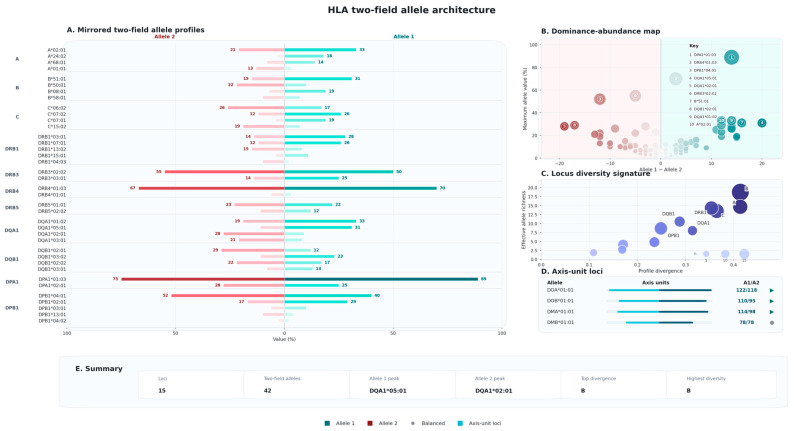
HLA two-field allele architecture across HLA loci. (**A**) Mirrored two-field allele profiles showing Allele 1 and Allele 2 call-position values for selected two-field alleles across Class I and Class II HLA loci. Values are displayed on a percentage scale for the main mirrored profile panel. (**B**) Dominance–abundance map summarizing both the direction of Allele 1–Allele 2 separation and the maximum allele value. (**C**) Locus diversity signature showing profile divergence and effective allele richness by locus. (**D**) Axis-unit loci showing DOA*01:01, DOB*01:01, DMA*01:01, and DMB*01:01 values outside the main percentage-scale display. (**E**) Summary panel showing the number of loci, total two-field entries, Allele 1 and Allele 2 peaks, top-divergence locus, and highest-diversity locus. Allele 1 and Allele 2 represent genotyping call positions and do not imply parental origin or phased inheritance.

The distribution of two-field HLA alleles was compared across Allele 1 and Allele 2 call positions for each HLA locus (Figure 2A–E). The mirrored profile panel summarized 38 percentage-scale two-field allele entries across HLA-A, HLA-B, HLA-C, HLA-DRB1, HLA-DRB3, HLA-DRB4, HLA-DRB5, HLA-DQA1, HLA-DQB1, HLA-DPA1, and HLA-DPB1, together with four additional axis-unit loci, yielding 42 total two-field entries across 15 loci.

For Class I loci, HLA-A*02:01 showed the highest Allele 1 value among HLA-A two-field alleles (33 versus 21), followed by A*24:02 and A*68:01, whereas A*01:01 was higher in Allele 2. For HLA-B, B*51:01 showed the clearest Allele 1 predominance (31 versus 15), whereas B*50:01 was higher in Allele 2 (10 versus 22). B*08:01 also showed a higher Allele 1 value. For HLA-C, C*07:02 and C*07:01 were higher in Allele 1, whereas C*06:02 and C*15:02 were higher in Allele 2.

For Class II loci, DRB1*03:01 and DRB1*07:01 were higher in Allele 1, whereas DRB1*13:02 was higher in Allele 2. DRB3*02:02 showed a modest Allele 2 predominance (50 versus 55), while DRB3*03:01 was higher in Allele 1. DRB4*01:03 showed high values in both call positions and was slightly higher in Allele 1 (70 versus 67). DRB5*01:01 was nearly balanced, whereas DRB5*02:02 was higher in Allele 1.

For HLA-DQ and HLA-DP two-field alleles, DQA1*01:02 and DQA1*05:01 were higher in Allele 1, while DQA1*02:01 and DQA1*03:01 were higher in Allele 2. DQB1*02:01 and DQB1*02:02 were higher in Allele 2, whereas DQB1*03:02 and DQB1*03:01 were higher in Allele 1. DPA1*01:03 showed the highest value in the mirrored profile panel and was higher in Allele 1 (89 versus 75), while DPA1*02:01 was slightly higher in Allele 2 (25 versus 28). For HLA-DPB1, DPB1*04:01 was higher in Allele 2 (40 versus 52), whereas DPB1*02:01 was higher in Allele 1 (29 versus 17).

The dominance–abundance map identified DPA1*01:03, DRB4*01:03, DPB1*04:01, DQA1*05:01, DQA1*02:01, DRB3*02:02, B*51:01, DQB1*02:01, DQA1*01:02, and A*02:01 as the most prominent two-field alleles when both dominance direction and abundance were considered. The locus diversity signature showed HLA-B as the locus with both the highest diversity and the top divergence signal. For the axis-unit loci, DOA*01:01, DOB*01:01, and DMA*01:01 showed higher Allele 1 axis-unit values, whereas DMB*01:01 was balanced across the two call positions. Additional two-field allele visualizations are provided in Supplementary Figures S6–S10, including the full mirrored profiles, class-specific mirrored profiles, dominance–abundance map, locus-diversity detail, original-style bar plot audit, and axis-unit detail.

### 3.5. Distribution of HLA Class I and II Allele Groups Between COVID-19 Recovered and Deceased Patients

In the comparison of HLA Class I and II allele groups between COVID-19 recovered patients (*n* = 102) and deceased patients (*n* = 21), several statistically significant call-position-specific differences were observed (Table 3). Among HLA-B Allele 1 calls, B*15 and B*53 were significantly more frequent among deceased patients, whereas B*51 was significantly more frequent among recovered patients. Among HLA-C Allele 2 calls, C*06 was significantly more frequent among deceased patients, while C*15 was detected only among recovered patients. Regarding HLA Class II loci, DRB1*10 and DQB1*05 in Allele 1 were significantly more frequent among deceased patients, whereas DQB1*03 in Allele 1 was significantly more frequent among recovered patients.

### 3.6. Distribution of HLA Class I and II Allele Groups Between COVID-19 Clinical Severity Groups

Analysis of selected HLA Class I and II allele groups showed call-position-specific differences across COVID-19 clinical severity comparisons (Table 4). HLA-C*15 in Allele 2 was significantly more frequent in Stage A patients than in patients with Stage B+C+D disease (50.0% versus 17.7%, *p* = 0.029). HLA-C*06 in Allele 1 was significantly more frequent in Stage A+B patients than in Stage C+D patients (23.8% versus 8.6%, *p* = 0.028). Among HLA-B allele groups, B*14 showed an any-call signal and was observed only in Stage D patients (10.5% versus 0.0%, *p* = 0.023), while B*39 and B*53 in Allele 2 were more frequent in Stage D patients than in Stage A+B+C patients (15.8% versus 2.9% for both, *p* = 0.047). For HLA Class II, DQB1*03 in Allele 1 was significantly more frequent among Stage A+B+C patients than among Stage D patients (44.2% versus 15.8%, *p* = 0.022).

### 3.7. Vaccination Status According to COVID-19 Survival and Clinical Severity Groups

Vaccination status was available for all 123 patients (Table 5). Overall, 45 patients (36.6%) were vaccinated and 78 (63.4%) were unvaccinated. Vaccination status did not differ significantly between recovered and deceased patients (37.3% versus 33.3% vaccinated, *p* = 0.808). Across clinical severity stages, vaccination frequencies were 50.0% in Stage A, 31.3% in Stage B, 43.5% in Stage C, and 15.8% in Stage D; the overall four-stage comparison was not statistically significant (*p* = 0.111).

Exploratory interaction analyses showed no significant age–sex, BMI–sex, or age–BMI interaction for mortality or Stage D disease. For the Stage C+D versus Stage A+B comparison, the age–sex interaction reached statistical significance (*p* = 0.033), whereas BMI–sex (*p* = 0.074) and age–BMI (*p* = 0.271) interactions were not statistically significant; these exploratory results are presented in Supplementary Table S29.

Complete HLA allele-group distributions by allele-call position are provided in Supplementary Tables S1–S28, with the any-call HLA-B*14 severity comparison shown in Supplementary Table S23A. Exploratory age–sex–BMI interaction analyses are presented in Supplementary Table S29.

## 4. Discussion

In this prospective Saudi cohort of laboratory-confirmed COVID-19 patients, we performed comprehensive HLA genotyping to evaluate HLA allele-group differences according to survival outcome and clinical severity. In survival analyses, the significant HLA associations were call-position specific. HLA Class I allele groups B*15 and B*53 in Allele 1 and C*06 in Allele 2, together with HLA Class II allele groups DRB1*10 and DQB1*05 in Allele 1, were more frequent among deceased patients. In contrast, B*51 in Allele 1, C*15 in Allele 2, and DQB1*03 in Allele 1 were more frequent among recovered patients, suggesting a possible association with favorable clinical outcome in this cohort. In severity analyses, the significant HLA associations were also call-position specific. HLA Class I allele group B*14 was observed as an any-call signal, whereas B*39 and B*53 in Allele 2 were more frequent among Stage D patients. In contrast, the HLA Class II allele group DQB1*03 in Allele 1 was more frequent among patients with non-critical disease.

The additional vaccination-status analysis showed no significant difference in vaccination frequency between recovered and deceased patients or across the four clinical severity stages. Therefore, the observed HLA survival and severity patterns were not accompanied by a major crude imbalance in vaccination status in this cohort. However, vaccination timing, vaccine type, antibody response, and interval between vaccination and infection were not evaluated in detail; therefore, residual confounding by vaccination-related factors cannot be fully excluded.

In our cohort, HLA-DPB1 represented the highest proportion of HLA calls. HLA-DPB1 encodes the beta chain of the HLA-DP Class II molecule, which contributes to antigen presentation to CD4+ T cells and may therefore influence antiviral immune responses. The HLA-DPB1 locus is highly polymorphic, and different allelic variants may contribute to population-specific differences in immune recognition and clinical outcomes. In relation to COVID-19, evidence regarding HLA-DPB1 and broader HLA genotype effects remains mixed [20,21,22]. Farias et al. reported that HLA-DPB1*13:01 was associated with protection against severe COVID-19 infection [20]. In contrast, Suslova et al. reported that the HLA-DPB1 rs9277534 A/G polymorphism was associated with increased COVID-19 risk and reduced HLA-DPB1 protein expression [21]. Other cohort-level analyses have reported minimal observed impact of HLA genotype on hospitalization and severity [22]. These contrasting findings suggest that HLA effects may be allele-specific, population-dependent, and influenced by regulatory variation. Therefore, the high representation of HLA-DPB1 calls in the present cohort supports the relevance of HLA typing for understanding population-level immune diversity, but it should not be interpreted as direct evidence of either susceptibility or protection without allele-specific association analysis.

Research studies have documented that HLA Class I and Class II alleles may be associated with COVID-19 disease severity and clinical outcomes. As noted earlier, HLA molecules play a pivotal role in generating effective immune responses during SARS-CoV-2 infection. In the present survival analysis, HLA Class I allele groups B*15 and B*53 in Allele 1 and C*06 in Allele 2, together with HLA Class II allele groups DRB1*10 and DQB1*05 in Allele 1, were more frequent among deceased patients. Conversely, HLA Class I allele groups B*51 in Allele 1 and C*15 in Allele 2 and the HLA Class II allele group DQB1*03 in Allele 1 were more frequent among recovered patients. These findings suggest that both Class I and Class II HLA loci may be associated with survival patterns in Saudi patients with COVID-19.

Several studies have reported associations between HLA Class I alleles and COVID-19 mortality or poor clinical outcomes [23,24,25,26,27]. Sakuraba et al. reported that HLA-A*01, HLA-B*07, HLA-B*08, HLA-B*44, and HLA-C*05 were associated with COVID-19 mortality in univariable logistic regression, whereas only HLA-C*05 remained significant in multivariable regression [23]. In Saudi Arabia, Naemi et al. reported a higher frequency of HLA-B*51 among South Asian COVID-19 patients who died compared with milder cases, whereas HLA-B*35 was more frequent in milder cases than in fatal cases [24]. A Russian cohort study reported that HLA-A*01:01 may be linked with increased susceptibility to COVID-19-associated death, and that HLA-A*01:01 homozygosity was associated with increased risk of early death before 60 years of age [25]. Tomita et al. performed an in silico analysis and reported an association between HLA-A*02:01 frequency and population-level COVID-19 case and mortality rates [28]. In Egyptian patients with COVID-19, Abdelhafiz et al. reported that HLA-B*41, HLA-B*42, HLA-C*16, and HLA-C*17 were associated with severe COVID-19 and poor survival, whereas HLA-B*15, HLA-C*07, and HLA-C*12 were associated with comparatively better survival; in regression analysis, HLA-B*15 was the only allele predicting better survival [26]. A preliminary study from Saudi Arabia reported higher frequencies of HLA-A*01, HLA-B*56, and HLA-C*01 among COVID-19 patients compared with controls, and higher frequencies of HLA-A*03 and HLA-C*06 among fatal COVID-19 cases [24]. Another study from Spain reported associations of HLA-A*11 and HLA-C*01 with COVID-19 death after controlling for clinical confounders such as SOFA and APACHE-II scores [27].

For HLA Class II, DRB1*10 and DQB1*05 in Allele 1 were more frequent among deceased patients, whereas DQB1*03 in Allele 1 was more frequent among recovered patients. These findings support the possibility that Class II antigen-presentation pathways may contribute to differences in COVID-19 survival outcomes. In an Italian cohort, Amoroso et al. reported that HLA-DRB1*08 was associated with approximately three-fold higher odds of COVID-19-associated mortality [29]. In contrast, Romero-López et al. reported a negative correlation between HLA-DRB1*01 and in-hospital mortality in Mexican patients with COVID-19 [30]. More recently, Abolnezhadian et al. reported that HLA-DRB1*11:01 was more frequent in fatal COVID-19 cases, whereas several other alleles were associated with protection [31]. Lorente et al. reported higher mortality among patients with HLA-DQB1*04 in a Spanish cohort; this allele has also been linked with unfavorable outcomes in other infectious diseases, including hepatitis B virus infection and active pulmonary tuberculosis [27].

We also analyzed the distribution of HLA Class I and Class II allele groups in relation to COVID-19 clinical severity: Stage A (asymptomatic infection), Stage B (mild infection), Stage C (moderate infection), and Stage D (severe infection). The severity-based findings were call-position specific. HLA-C*15 in Allele 2 was more frequent in Stage A than in Stage B+C+D disease, whereas HLA-C*06 in Allele 1 was more frequent in Stage A+B than in Stage C+D disease. In the Stage A+B+C versus Stage D comparison, HLA-B*14 showed an any-call signal, and HLA-B*39 and HLA-B*53 in Allele 2 were more frequent among Stage D patients. In contrast, the HLA Class II allele group DQB1*03 in Allele 1 was more frequent among patients with non-critical disease.

Several studies have highlighted the potential role of HLA alleles in modulating COVID-19 disease severity. HLA-B*07 has been associated with increased risk of severe COVID-19, whereas HLA-B*27 and HLA-C*12:02 were reported at higher frequencies among patients with mild COVID-19, suggesting a possible protective association [32]. In a Romanian cohort, Vică et al. reported that HLA-B*27 and HLA-B*50 were associated with a higher propensity for severe COVID-19, whereas HLA-A*33 and HLA-C*15 were suggested to have protective associations. The same study also reported that HLA-A*03 and HLA-DQB1*02 were associated with protection against COVID-19 disease severity [6]. Augusto et al. confirmed a robust association between HLA-B*15:01 and asymptomatic COVID-19 infection, supported by experimental evidence showing pre-existing T-cell reactivity to an immunodominant SARS-CoV-2 peptide in HLA-B*15:01-positive individuals [33]. Lobato-Martinez et al. identified HLA-C*05:01 and HLA-DQB1*02:02 as protective against COVID-19-associated hospital admission, whereas HLA-DQA1*05:01 was associated with increased risk of ICU admission or mortality [18]. Langton et al. investigated HLA Class I and Class II genes in 147 European individuals with diverse COVID-19 outcomes and reported differences involving HLA-DRB1*04:01, which was more frequent among asymptomatic patients than among severe cases. They further noted a higher frequency of this allele in Northwestern European populations, supporting the possibility that population-specific HLA distributions may influence COVID-19 outcomes [34]. In Vietnamese patients with COVID-19, several allelic differences were also reported: HLA-F*01:01, HLA-F*01:03, HLA-DPA1*01:03, HLA-DPA1*02:01, HLA-DPB1*04:01, HLA-DQA1*01:02, and HLA-DQB1*05:02 were more frequent among patients with severe COVID-19, whereas HLA-DOB*01:01, HLA-DRB1*05:01, and HLA-DRB1*09:01 were more frequent among mild cases [19].

We also explored whether age, sex, and BMI interacted in relation to mortality and clinical severity. No significant age–sex, BMI–sex, or age–BMI interaction was observed for mortality or Stage D disease. An age–sex interaction was observed for the Stage C+D versus Stage A+B comparison, but this finding should be considered exploratory because of the limited cohort size and the small number of patients in some severity strata. Larger cohorts are needed to confirm whether demographic and anthropometric factors modify HLA-associated COVID-19 outcomes.

Several limitations should be considered when interpreting these findings. First, the study cohort was relatively small, which reduces statistical power, widens confidence intervals, and increases the possibility of both type I and type II error, particularly for low-frequency HLA allele groups [35]. Second, the study was conducted at a single center, which may limit geographic and population diversity and therefore restrict the generalizability of the findings. HLA allele distributions vary substantially across populations, and these differences may influence COVID-19 immune-response patterns [36]. Third, the study included hospitalized patients and did not include a non-hospitalized SARS-CoV-2-positive comparator group. Therefore, the findings should be interpreted as associations with survival and severity within the enrolled COVID-19 cohort rather than as case–control evidence for susceptibility to SARS-CoV-2 infection. Fourth, potential selection bias and unmeasured confounding may have influenced the observed associations [37]. Clinical outcomes in COVID-19 can be affected by socioeconomic background [38], co-existing diseases [39], lifestyle factors [40], and vaccination-related factors [41]. Although vaccination status was compared between outcome and severity groups, detailed information on vaccine type, vaccination timing, antibody response, and interval between vaccination and infection was not incorporated into adjusted HLA models. Finally, rare HLA allele groups may have been underrepresented, limiting the ability to detect associations involving low-frequency alleles. Larger multicenter studies with non-hospitalized comparator groups, detailed vaccination data, and adjusted modeling are needed to confirm these findings.

## 5. Conclusions

In conclusion, this prospective study suggests that selected HLA Class I and Class II allele groups may be associated with COVID-19 survival and clinical severity patterns in Saudi patients. In survival-based comparisons, B*15 and B*53 in Allele 1 and C*06 in Allele 2, together with DRB1*10 and DQB1*05 in Allele 1, were more frequent among deceased patients, whereas B*51 in Allele 1, C*15 in Allele 2, and DQB1*03 in Allele 1 were more frequent among recovered patients. In severity-based comparisons, C*15 in Allele 2 was more frequent in Stage A than in Stage B+C+D disease, C*06 in Allele 1 was more frequent in Stage A+B than in Stage C+D disease, B*14 showed an any-call signal, and B*39 and B*53 in Allele 2 were more frequent among Stage D patients; DQB1*03 in Allele 1 was more frequent among patients with non-critical disease. The allele-position and two-field analyses further showed distinct Allele 1–Allele 2 call-position architectures across HLA loci; however, these call-position patterns should not be interpreted as parental inheritance because phasing was not performed. The findings should be interpreted cautiously because of the small single-center cohort, the absence of a non-hospitalized SARS-CoV-2-positive comparator group, the limited representation of low-frequency HLA allele groups, and the possibility of residual confounding. Future multicenter studies should validate these associations using larger cohorts, detailed vaccination information, and adjusted analyses.

## Figures and Tables

**Table 1:** Demographic and Clinical Characteristics of COVID-19 Patients According to Clinical Outcome Groups.

Characteristics	Clinical Outcome Groups		*p* Value
	Deceased (*n* = 21)	Recovered (*n* = 102)	
Sex, *n* (%) *			
Male	13 (61.90)	47 (46.08)	0.186
Female	8 (38.10)	55 (53.92)	
**Age, Median (25th–75th)** ^†^	60 (48.50–71.50)	57 (39.0–69.0)	0.279
**BMI, Median (25th–75th)** ^†^	26.5 (23.55–31.20)	27.75 (22.55–32.30)	0.967
**Thrombosis, *n* (%)** *			
Yes	1 (4.76)	5 (4.90)	1.000
No	20 (95.24)	97 (95.10)	
**WBC, Mean ± SD** *	7.77 ± 6.85	7.54 ± 5.80	0.875
**Creatinine, Mean ± SD** *	117.76 ± 75.55	154.14 ± 276.19	0.551
**Coagulation/Hemostatic Markers**			
Platelet, Median (25th–75th) ^†^	186 (134.00–250.50)	211.5 (130.50–257.25)	0.721
INR, Mean ± SD *	1.25 ± 0.39	1.49 ± 2.01	0.596
PT, Mean ± SD *	16.99 ± 5.01	17.58 ± 6.71	0.706
PTT, Mean ± SD *	40.62 ± 7.73	41.07 ± 11.51	0.863
D-Dimer, Median (25th–75th) ^†^	1.13 (0.78–3.45)	1.1 (0.56–2.43)	0.967
Fibrinogen, Mean ± SD *	5.13 ± 2.08	4.67 ± 1.43	0.346
**ICU Admission, *n* (%)** *			
Yes	20 (95.24)	52 (50.98)	**0.0001**
No	1 (4.76)	50 (49.02)	
**Length of hospital stay, Mean ± SD** *	38.19 ± 25.39	21.41 ± 38.90	0.061

Abbreviations: COVID-19, coronavirus disease 2019; BMI, body mass index; SD, standard deviation; WBC, white blood cell count; INR, international normalized ratio; PT, prothrombin time; PTT, partial thromboplastin time; ICU, intensive care unit. * Chi-square/Fisher’s exact and Independent Samples *t*-tests. ^†^ Nonparametric (Mann–Whitney U) tests. Bold indicates statistically significant *p* values.

**Table 2:** Demographic and Clinical Characteristics of COVID-19 Patients According to Clinical Severity Groups.

Characteristics	COVID-19 Clinical Severity Groups				*p* (A vs. B+C+D)	*p* (A+B vs. C+D)	*p* (A+B+C vs. D)
	A (Asymptomatic) (*n* = 10)	B (Mild) (*n* = 32)	C (Moderate) (*n* = 62)	D (Severe) (*n* = 19)			
**Sex, *n* (%)** *							
Male	5 (50)	15 (46.88)	31 (50)	9 (47.37)	1.00	0.853	0.893
Female	5 (50)	17 (53.13)	31 (50)	10 (52.63)			
**Age, Median (25th–75th) ^†^**	45.5 (22.00–64.25)	42.5 (22.50–57.75)	59.5 (45.75–70.50)	70 (49.00–79.00)	0.803	**0.001**	0.11
**BMI, Median (25th–75th) ^†^**	25.55 (18.30–30.45)	26.75 (23.15–30.50)	27.9 (22.60–33.50)	28.7 (24.50–32.50)	0.762	0.376	0.30
**Thrombosis, *n* (%)** *							
Yes	0 (0.0)	4 (12.50)	0 (0.0)	2 (10.53)	1.00	0.179	0.232
No	10 (100)	28 (87.50)	62 (100)	17 (89.47)			
**WBC, Mean ± SD** *	9.04 ± 5.19	7.12 ± 4.02	7.00 ± 6.45	9.48 ± 7.22	0.42	0.996	0.213
**Creatinine, Mean ± SD** *	271.20 ± 298.65	106.84 ± 121.70	156.29 ± 318.30	124.94 ± 99.79	0.109	0.951	0.669
**Coagulation/Hemostatic Markers**							
Platelet, Median (25th–75th) ^†^	227.5 (149.00–342.75)	226 (142.50–261.75)	193.5 (123.50–237.25)	203 (143.00–237.00)	0.681	**0.022**	0.167
INR, Mean ± SD *	3.15 ± 6.27	1.43 ± 0.705	1.27 ± 0.446	1.15 ± 0.167	0.376	0.218	0.45
PT, Mean ± SD *	17.04 ± 4.40	19.08 ± 9.04	17.28 ± 5.90	15.64 ± 2.03	0.822	0.166	**0.009**
PTT, Mean ± SD *	37.55 ± 19.22	40.94 ± 10.86	41.54 ± 9.98	41.14 ± 8.75	0.30	0.529	0.95
D-Dimer, Median (25th–75th) ^†^	1.55 (0.83–2.74)	0.795 (0.50–2.81)	1.075 (0.64–2.12)	1.4 (0.70–3.68)	0.309	0.613	0.30
Fibrinogen, Mean ± SD *	3.96 ± 1.53	4.26 ± 1.68	4.99 ± 1.32	5.18 ± 1.84	0.099	**0.004**	0.189
**ICU Admission, *n* (%)** *							
Yes	3 (30.0)	14 (43.75)	36 (58.06)	19 (100)	0.091	**0.003**	**<0.0001**
No	7 (70.0)	18 (56.25)	26 (41.94)	0 (0.0)			
**Length of Stay, Mean ± SD** *	50.5 ± 110.6	13.65 ± 15.72	20.93 ± 19.89	39.26 ± 27.19	0.436	0.695	0.057

Abbreviations: COVID-19, coronavirus disease 2019; BMI, body mass index; SD, standard deviation; WBC, white blood cell count; INR, international normalized ratio; PT, prothrombin time; PTT, partial thromboplastin time; ICU, intensive care unit. * Chi-square/Fisher’s exact and Independent Samples *t*-tests. ^†^ Nonparametric (Mann–Whitney U) tests. Bold indicates statistically significant *p* values.

**Table 3:** Distribution of Statistically Significant HLA Class I and II Allele Groups between COVID-19 Recovered and Deceased Patients by Allele-Call Position.

Characteristics	Allele-Call Position	Survival Groups		*p* Value
		Recovered (*n* = 102), *n* (%)	Deceased (*n* = 21), *n* (%)	
**HLA-B**				
B*15	Allele 1	1 (1.0)	3 (14.3)	**0.016**
B*51	Allele 1	31 (30.4)	0 (0.0)	**0.002**
B*53	Allele 1	0 (0.0)	3 (14.3)	**0.004**
**HLA-C**				
C*06	Allele 2	19 (18.6)	9 (42.9)	**0.023**
C*15	Allele 2	25 (24.5)	0 (0.0)	**0.007**
**HLA-DRB1**				
DRB1*10	Allele 1	5 (4.9)	4 (19.0)	**0.045**
**HLA-DQB1**				
DQB1*03	Allele 1	46 (45.1)	3 (14.3)	**0.013**
DQB1*05	Allele 1	12 (11.8)	7 (33.3)	**0.021**

**Abbreviations:** HLA, human leukocyte antigen; COVID-19, coronavirus disease 2019. **Note:** HLA-B and HLA-C are Class I loci, whereas HLA-DRB1 and HLA-DQB1 are Class II loci. Allele 1 and Allele 2 indicate genotyping call positions and do not imply parental origin or phased inheritance. *p* values were calculated using Fisher’s exact test. Bold indicates statistically significant *p* values.

**Table 4:** Distribution of Statistically Significant HLA Class I and II Allele Groups between COVID-19 Clinical Severity Groups by Allele-Call Position.

Characteristics	Allele-Call Position	COVID-19 Clinical Severity Groups		*p* Value
		A (*n* = 10)	B+C+D (*n* = 113)	
**HLA-C**				
C*15	Allele 2	5 (50.0)	20 (17.7)	**0.029**
		**A+B** **(*n* = 42)**	**C+D** **(*n* = 81)**	
**HLA-C**				
C*06	Allele 1	10 (23.8)	7 (8.6)	**0.028**
		**A+B+C** **(*n* = 104)**	**D** **(*n* = 19)**	
**HLA-B**				
B*14	Any allele-call	0 (0.0)	2 (10.5)	**0.023**
B*39	Allele 2	3 (2.9)	3 (15.8)	**0.047**
B*53	Allele 2	3 (2.9)	3 (15.8)	**0.047**
**HLA-DQB1**				
DQB1*03	Allele 1	46 (44.2)	3 (15.8)	**0.022**

Abbreviations: HLA, human leukocyte antigen; COVID-19, coronavirus disease 2019. **Note:** HLA-B and HLA-C are Class I loci, whereas HLA-DQB1 is a Class II locus. Allele 1 and Allele 2 indicate genotyping call positions and do not imply parental origin or phased inheritance. Any allele-call indicates detection in either Allele 1 or Allele 2. *p* values were calculated using Fisher’s exact test. Bold indicates statistically significant *p* values.

**Table 5:** Distribution of Vaccination Status According to COVID-19 Survival and Clinical Severity Groups.

Group Comparison	Clinical Group	Vaccinated *n* (%)	Unvaccinated *n* (%)	*p* Value
Overall cohort	Total (*n* = 123)	45 (36.6)	78 (63.4)	—
Survival status	Recovered (*n* = 102)	38 (37.3)	64 (62.7)	0.808
	Deceased (*n* = 21)	7 (33.3)	14 (66.7)	
Clinical severity stage	Stage A (*n* = 10)	5 (50.0)	5 (50.0)	0.111
	Stage B (*n* = 32)	10 (31.3)	22 (68.8)	
	Stage C (*n* = 62)	27 (43.5)	35 (56.5)	
	Stage D (*n* = 19)	3 (15.8)	16 (84.2)	

Abbreviations: COVID-19, coronavirus disease 2019. Note: Vaccination status was available for all 123 patients. *p* values were calculated using Fisher’s exact test for survival status and Pearson’s chi-square test for the four-stage clinical severity comparison.

## Data Availability

The data supporting the findings of this study are available from the corresponding author upon reasonable request. Due to privacy and ethical restrictions, the dataset is not publicly available.

## Supplementary Materials

The following supporting information can be downloaded at: https://www.mdpi.com/article/10.3390/biomedicines14061220/s1, Supplementary Tables S1–S29 provide the complete HLA allele-group distributions, call-position-specific survival and severity comparisons, any-call HLA-B*14 severity comparison, and exploratory age–sex–BMI interaction analyses. Supplementary Figures S1–S5 provide extended allele-group visualizations supporting Figure 1, whereas Supplementary Figures S6–S10 provide extended two-field allele visualizations supporting Figure 2.

## Abbreviations

The following abbreviations are used in this manuscript:

KFSH&RC	King Faisal Specialist Hospital and Research Centre
COVID-19	Coronavirus disease 2019
PCR-SSO	Polymerase chain reaction-sequence specific oligonucleotide
MHC	Major histocompatibility complex
HLA	Human leukocyte antigen
MERS-CoV	Middle East Respiratory Syndrome Coronavirus
SARS-CoV	Severe Acute Respiratory Syndrome Coronavirus
ERC	Ethics Review Committee
ICU	Intensive care unit
PCR	Polymerase chain reaction
INR	International normalized ratio
PT	Prothrombin time
PTT	Partial thromboplastin time
SD	Standard deviation
IQR	Interquartile range
OR	Odds ratio
CI	Confidence interval
