# Supplementary material for: HLA Class I and II Variants as Potential Determinants of Clinical Severity and Mortality in Patients with COVID-19: A Prospective Study from Saudi Arabia
Source: Biomedicines. 2026 May 28;14(6):1220. doi: 10.3390/biomedicines14061220 (PMC13296798; doi:10.3390/biomedicines14061220)
Supplement: Supplementary file 1 [file biomedicines-14-01220-s001.zip › Supplementary Figure S1.pdf]

Full percentage rows

	Locus	Allele group	Allele 1	Value	Allele 2	Value	Δ	Score
Class I	HLA-A	A*02		43		22		+21
		A*24		19		4		+15
		A*01		3		15		-12
		A*68		15		11		+4
		A*03		3		13		-10
		A*30		0		13		-13
		A*23		9		5		+4
		A*33		2		9		-7
		A*31		8		8		0
		A*26		5		6		-1
		A*32		4		5		-1
		A*74		4		2		+2
		A*11		3		3		0
		A*29		2		3		-1
		A*34		1		2		-1
		A*66		2		1		+1
		A*36		0		1		-1
	HLA-B	B*51		31		16		+15
		B*50		10		22		-12
		B*08		19		7		+12
		B*15		4		16		-12
		B*58		9		11		-2
		B*35		8		7		+1
		B*41		7		1		+6
		B*57		0		7		-7
		B*07		6		6		0
		B*39		4		6		-2
		B*53		3		6		-3
		B*44		4		1		+3
		B*40		3		3		0
		B*47		1		3		-2
		B*13		2		0		+2
		B*14		2		2		0
		B*18		2		0		+2
		B*37		2		1		+1
		B*49		2		2		0
		B*52		2		0		+2
		B*55		0		2		-2
		B*73		0		2		-2
		B*27		0		1		-1
		B*38		1		0		+1
		B*42		1		1		0
	HLA-C	C*07		48		20		+28
		C*06		17		28		-11
		C*15		8		25		-17
		C*03		3		18		-15
		C*04		17		9		+8
		C*14		8		3		+5
		C*17		3		8		-5
		C*16		5		1		+4
		C*01		4		1		+3
		C*12		4		3		+1
		C*08		3		3		0
		C*02		2		2		0
		C*05		1		1		0
		C*18		0		1		-1
Class II	HLA-DRB1	DRB1*04		10		32		-22
		DRB1*03		30		14		+16
		DRB1*07		26		12		+14
		DRB1*13		16		22		-6
		DRB1*11		7		19		-12
		DRB1*15		16		6		+10
		DRB1*10		9		4		+5
		DRB1*16		2		6		-4
		DRB1*01		4		1		+3
		DRB1*08		0		3		-3
		DRB1*09		1		2		-1
		DRB1*12		2		0		+2
		DRB1*14		0		2		-2
	HLA-DRB3	DRB3*02		55		62		-7
		DRB3*03		27		18		+9
		DRB3*01		12		14		-2
	HLA-DRB4	DRB4*01		81		81		0
	HLA-DRB5	DRB5*01		32		32		0
		DRB5*02		13		13		0
	HLA-DQA1	DQA1*01		53		39		+14
		DQA1*05		46		22		+24
		DQA1*03		15		30		-15
		DQA1*02		9		28		-19
		DQA1*04		0		2		-2
		DQA1*06		0		2		-2
	HLA-DQB1	DQB1*02		29		51		-22
		DQB1*03		49		23		+26
		DQB1*06		24		33		-9
		DQB1*05		19		16		+3
		DQB1*04		2		0		+2
	HLA-DPA1	DPA1*01		94		80		+14
		DPA1*02		28		41		-13
		DPA1*03		1		2		-1
	HLA-DPB1	DPB1*04		42		55		-13
		DPB1*02		29		17		+12
		DPB1*03		10		6		+4
		DPB1*13		4		10		-6
		DPB1*01		8		3		+5
		DPB1*104		5		7		-2
		DPB1*17		7		4		+3
		DPB1*14		4		3		+1
		DPB1*26		2		3		-1
		DPB1*30		1		3		-2
		DPB1*05		2		2		0
		DPB1*10		2		0		+2
		DPB1*105		2		1		+1
		DPB1*15		2		0		+2
		DPB1*834		2		2		0
		DPB1*09		0		1		-1
		DPB1*11		0		1		-1
		DPB1*1286		0		1		-1
		DPB1*131		0		1		-1
		DPB1*23		1		0		+1
		DPB1*417		0		1		-1
		DPB1*49		0		1		-1
		DPB1*519		0		1		-1
