# Supplementary material for: HLA Class I and II Variants as Potential Determinants of Clinical Severity and Mortality in Patients with COVID-19: A Prospective Study from Saudi Arabia
Source: Biomedicines. 2026 May 28;14(6):1220. doi: 10.3390/biomedicines14061220 (PMC13296798; doi:10.3390/biomedicines14061220)
Supplement: Supplementary file 1 [file biomedicines-14-01220-s001.zip › Supplementary Figure S4.pdf]

Locus signature

Locus	Top allele	Profile divergence	Dom.
**HLA-A**	A*02	0.42	
**HLA-B**	B*51	0.42	
**HLA-DRB1**	DRB1*04	0.39	
**HLA-C**	C*07	0.38	
**HLA-DQA1**	DQA1*01	0.30	
**HLA-DPB1**	DPB1*04	0.30	
**HLA-DQB1**	DQB1*02	0.24	
**HLA-DPA1**	DPA1*01	0.11	
**HLA-DRB3**	DRB3*02	0.10	
**HLA-DRB5**	DRB5*01	0.00	
**HLA-DRB4**	DRB4*01	0.00	
**HLA-DOA**	DOA*01	0.00	
**HLA-DOB**	DOB*01	0.00	
**HLA-DMA**	DMA*01	0.00	
**HLA-DMB**	DMB*01	0.00	
