# Supplementary material for: HLA Class I and II Variants as Potential Determinants of Clinical Severity and Mortality in Patients with COVID-19: A Prospective Study from Saudi Arabia
Source: Biomedicines. 2026 May 28;14(6):1220. doi: 10.3390/biomedicines14061220 (PMC13296798; doi:10.3390/biomedicines14061220)
Supplement: Supplementary file 1 [file biomedicines-14-01220-s001.zip › Supplementary Figure S5.pdf]

Global summary

C*07 +28	DRB1*04 -22	HLA-A	15	44	3 / 12
**Allele 1 peak**	**Allele 2 peak**	**Top divergence**	**Loci**	**Groups**	**Class split**
HLA-C	HLA-DRB1	0.42	displayed	main panel	I / II
