# Supplementary material for: HLA Class I and II Variants as Potential Determinants of Clinical Severity and Mortality in Patients with COVID-19: A Prospective Study from Saudi Arabia
Source: Biomedicines. 2026 May 28;14(6):1220. doi: 10.3390/biomedicines14061220 (PMC13296798; doi:10.3390/biomedicines14061220)

Supplementary Figure 7 - Figure 2 full dominance-abundance map

Full dominance-abundance map

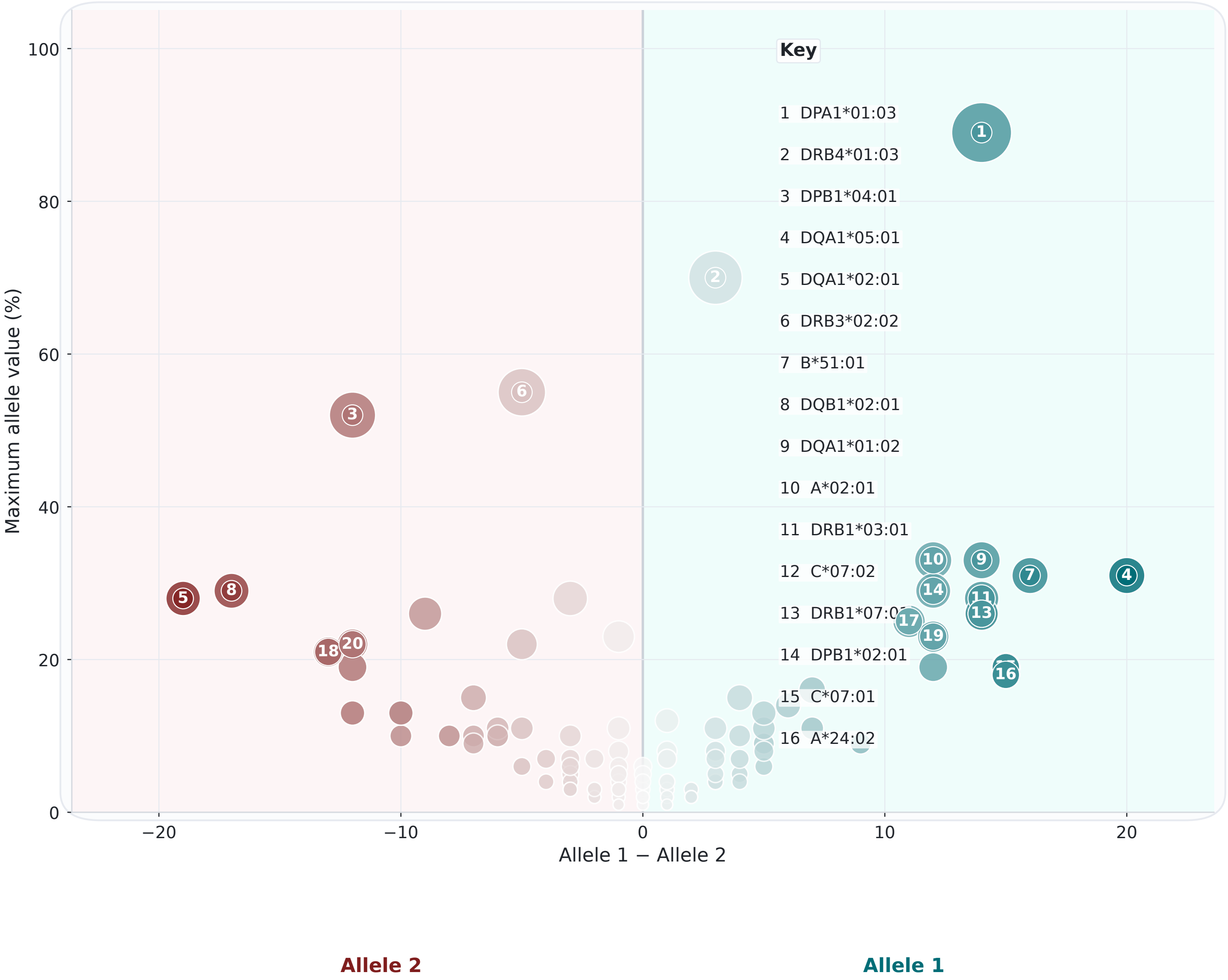

Supplement: Supplementary file 1 [file biomedicines-14-01220-s001.zip › Supplementary Figure S7.pdf]
