# Supplementary material for: HLA Class I and II Variants as Potential Determinants of Clinical Severity and Mortality in Patients with COVID-19: A Prospective Study from Saudi Arabia
Source: Biomedicines. 2026 May 28;14(6):1220. doi: 10.3390/biomedicines14061220 (PMC13296798; doi:10.3390/biomedicines14061220)
Supplement: Supplementary file 1 [file biomedicines-14-01220-s001.zip › Supplementary Tables.pdf]

Supplementary Table S1. Distribution of HLA-A Allele 2 Allele Groups between COVID-19 Recovered and Deceased Patients

Allele group	Recovered (n=102) n (%)	Deceased (n=21) n (%)	*P* Value
**A*01**	14 (13.7)	1 (4.8)	0.46
**A*02**	19 (18.6)	3 (14.3)	0.763
**A*03**	9 (8.8)	4 (19.0)	0.23
**A*11**	3 (2.9)	0 (0.0)	1
**A*23**	4 (3.9)	1 (4.8)	1
**A*24**	3 (2.9)	1 (4.8)	0.532
**A*26**	4 (3.9)	2 (9.5)	0.27
**A*29**	3 (2.9)	0 (0.0)	1
**A*30**	10 (9.8)	3 (14.3)	0.46
**A*31**	7 (6.9)	1 (4.8)	1
**A*32**	5 (4.9)	0 (0.0)	0.59
**A*33**	9 (8.8)	0 (0.0)	0.36
**A*34**	2 (2.0)	0 (0.0)	1
**A*36**	0 (0.0)	1 (4.8)	0.17
**A*66**	1 (1.0)	0 (0.0)	1
**A*68**	7 (6.9)	4 (19.0)	0.093
**A*74**	2 (2.0)	0 (0.0)	1

Abbreviations: HLA, human leukocyte antigen; COVID-19, coronavirus disease 2019.
Note: Allele 1 and Allele 2 indicate genotyping call positions and do not imply parental origin or phased inheritance. P values were calculated using Fisher's exact test.

Supplementary Table S2. Distribution of HLA-B Allele 1 Allele Groups between COVID-19 Recovered and Deceased Patients

**Allele group**	**Recovered (n=102) n (%)**	**Deceased (n=21) n (%)**	***P* Value**
**B*07**	6 (5.9)	0 (0.0)	0.588
**B*08**	13 (12.7)	6 (28.6)	0.094
**B*13**	2 (2.0)	0 (0.0)	1
**B*14**	1 (1.0)	1 (4.8)	0.313
**B*15**	1 (1.0)	3 (14.3)	0.016
**B*18**	2 (2.0)	0 (0.0)	1
**B*35**	7 (6.9)	1 (4.8)	1
**B*37**	2 (2.0)	0 (0.0)	1
**B*38**	0 (0.0)	1 (4.8)	0.171
**B*39**	3 (2.9)	1 (4.8)	0.532
**B*40**	3 (2.9)	0 (0.0)	1
**B*41**	7 (6.9)	0 (0.0)	0.602
**B*42**	1 (1.0)	0 (0.0)	1
**B*44**	3 (2.9)	1 (4.8)	0.532
**B*47**	1 (1.0)	0 (0.0)	1
**B*49**	2 (2.0)	0 (0.0)	1
**B*50**	6 (5.9)	4 (19.0)	0.067
**B*51**	31 (30.4)	0 (0.0)	0.002
**B*52**	2 (2.0)	0 (0.0)	1
**B*53**	0 (0.0)	3 (14.3)	0.004
**B*58**	9 (8.8)	0 (0.0)	0.355

Abbreviations: HLA, human leukocyte antigen; COVID-19, coronavirus disease 2019.

Note: Allele 1 and Allele 2 indicate genotyping call positions and do not imply parental origin or phased inheritance. P values were calculated using Fisher's exact test.

Supplementary Table S3. Distribution of HLA-C Allele 2 Allele Groups between COVID-19 Recovered and Deceased Patients

Allele group	Recovered (n=102) n (%)	Deceased (n=21) n (%)	*P* Value
**C*01**	1 (1.0)	0 (0.0)	1
**C*02**	2 (2.0)	0 (0.0)	1
**C*03**	14 (13.7)	4 (19.0)	0.509
**C*04**	7 (6.9)	2 (9.5)	0.65
**C*05**	1 (1.0)	0 (0.0)	1
**C*06**	19 (18.6)	9 (42.9)	0.023
**C*07**	18 (17.6)	2 (9.5)	0.522
**C*08**	1 (1.0)	2 (9.5)	0.075
**C*12**	2 (2.0)	1 (4.8)	0.433
**C*14**	3 (2.9)	0 (0.0)	1
**C*15**	25 (24.5)	0 (0.0)	0.007
**C*16**	0 (0.0)	1 (4.8)	0.171
**C*17**	8 (7.8)	0 (0.0)	0.349
**C*18**	1 (1.0)	0 (0.0)	1

Abbreviations: HLA, human leukocyte antigen; COVID-19, coronavirus disease 2019.

Note: Allele 1 and Allele 2 indicate genotyping call positions and do not imply parental origin or phased inheritance. P values were calculated using Fisher's exact test.

Supplementary Table S4. Distribution of HLA-DRB1 Allele 1 Allele Groups between COVID-19 Recovered and Deceased Patients

**Allele group**	**Recovered (n=102)**<br>**n (%)**	**Deceased (n=21)**<br>**n (%)**	***P* Value**
**DRB1*01**	4 (3.9)	0 (0.0)	1
**DRB1*03**	23 (22.5)	7 (33.3)	0.295
**DRB1*04**	10 (9.8)	0 (0.0)	0.209
**DRB1*07**	22 (21.6)	4 (19.0)	1
**DRB1*09**	1 (1.0)	0 (0.0)	1
**DRB1*10**	5 (4.9)	4 (19.0)	0.045
**DRB1*11**	6 (5.9)	1 (4.8)	1
**DRB1*12**	2 (2.0)	0 (0.0)	1
**DRB1*13**	14 (13.7)	2 (9.5)	1
**DRB1*15**	14 (13.7)	2 (9.5)	1
**DRB1*16**	1 (1.0)	1 (4.8)	0.313

Abbreviations: HLA, human leukocyte antigen; COVID-19, coronavirus disease 2019.
Note: Allele 1 and Allele 2 indicate genotyping call positions and do not imply parental origin or phased inheritance. P values were calculated using Fisher's exact test.

Supplementary Table S5. Distribution of HLA-DRB1 Allele 2 Allele Groups between COVID-19 Recovered and Deceased Patients

Allele group	Recovered (n=102) n (%)	Deceased (n=21) n (%)	*P* Value
**DRB1*01**	1 (1.0)	0 (0.0)	1
**DRB1*03**	10 (9.8)	4 (19.0)	0.257
**DRB1*04**	27 (26.5)	5 (23.8)	1
**DRB1*07**	8 (7.8)	4 (19.0)	0.123
**DRB1*08**	3 (2.9)	0 (0.0)	1
**DRB1*09**	2 (2.0)	0 (0.0)	1
**DRB1*10**	3 (2.9)	1 (4.8)	0.532
**DRB1*11**	18 (17.6)	1 (4.8)	0.192
**DRB1*13**	18 (17.6)	4 (19.0)	1
**DRB1*14**	2 (2.0)	0 (0.0)	1
**DRB1*15**	6 (5.9)	0 (0.0)	0.588
**DRB1*16**	4 (3.9)	2 (9.5)	0.272

Abbreviations: HLA, human leukocyte antigen; COVID-19, coronavirus disease 2019.

Note: Allele 1 and Allele 2 indicate genotyping call positions and do not imply parental origin or phased inheritance. P values were calculated using Fisher's exact test.

Supplementary Table S6. Distribution of HLA-DQB1 Allele 1 Allele Groups between COVID-19 Recovered and Deceased Patients

**Allele group**	**Recovered (n=102) n (%)**	**Deceased (n=21) n (%)**	***P* Value**
**DQB1*02**	22 (21.6)	7 (33.3)	0.266
**DQB1*03**	46 (45.1)	3 (14.3)	0.013
**DQB1*04**	2 (2.0)	0 (0.0)	1
**DQB1*05**	12 (11.8)	7 (33.3)	0.021
**DQB1*06**	20 (19.6)	4 (19.0)	1

Abbreviations: HLA, human leukocyte antigen; COVID-19, coronavirus disease 2019.

Note: Allele 1 and Allele 2 indicate genotyping call positions and do not imply parental origin or phased inheritance. P values were calculated using Fisher's exact test.

Supplementary Table S7. Distribution of HLA-DQB1 Allele 2 Allele Groups between COVID-19 Recovered and Deceased Patients

**Allele group**	**Recovered (n=102) n (%)**	**Deceased (n=21) n (%)**	***P* Value**
**DQB1*02**	39 (38.2)	12 (57.1)	0.109
**DQB1*03**	20 (19.6)	3 (14.3)	0.762
**DQB1*05**	13 (12.7)	3 (14.3)	0.736
**DQB1*06**	30 (29.4)	3 (14.3)	0.154

Abbreviations: HLA, human leukocyte antigen; COVID-19, coronavirus disease 2019.

Note: Allele 1 and Allele 2 indicate genotyping call positions and do not imply parental origin or phased inheritance. P values were calculated using Fisher's exact test.

Supplementary Table S8. Distribution of HLA-A Allele 2 Allele Groups between COVID-19 Stage A and B+C+D

Allele group	Stage A (n=10) n (%)	Stage B+C+D (n=113) n (%)	*P* Value
**A*01**	2 (20.0)	13 (11.5)	0.35
**A*02**	4 (40.0)	18 (15.9)	0.08
**A*03**	2 (20.0)	11 (9.7)	0.29
**A*11**	0 (0.0)	3 (2.7)	1
**A*23**	0 (0.0)	5 (4.4)	1
**A*24**	0 (0.0)	4 (3.5)	1
**A*26**	0 (0.0)	6 (5.3)	1
**A*29**	1 (10.0)	2 (1.8)	0.23
**A*30**	0 (0.0)	13 (11.5)	0.598
**A*31**	1 (10.0)	7 (6.2)	0.5
**A*32**	0 (0.0)	5 (4.4)	1
**A*33**	0 (0.0)	9 (8.0)	1
**A*34**	0 (0.0)	2 (1.8)	1
**A*36**	0 (0.0)	1 (0.9)	1
**A*66**	0 (0.0)	1 (0.9)	1
**A*68**	0 (0.0)	11 (9.7)	0.597
**A*74**	0 (0.0)	2 (1.8)	1

Abbreviations: HLA, human leukocyte antigen; COVID-19, coronavirus disease 2019.
Note: COVID Stage A: asymptomatic infection; COVID Stage B: mild infection; COVID Stage C: moderate infection; COVID Stage D: severe infection. Allele 1 and Allele 2 indicate genotyping call positions and do not imply parental origin or phased inheritance. P values were calculated using Fisher's exact test.

Supplementary Table S9. Distribution of HLA-B Allele 1 Allele Groups between COVID-19 Stage A and B+C+D

Allele group	Stage A (n=10) n (%)	Stage B+C+D (n=113) n (%)	*P* Value
**B*07**	0 (0.0)	6 (5.3)	1
**B*08**	1 (10.0)	18 (15.9)	1
**B*13**	0 (0.0)	2 (1.8)	1
**B*14**	0 (0.0)	2 (1.8)	1
**B*15**	0 (0.0)	4 (3.5)	1
**B*18**	0 (0.0)	2 (1.8)	1
**B*35**	2 (20.0)	6 (5.3)	0.128
**B*37**	0 (0.0)	2 (1.8)	1
**B*38**	0 (0.0)	1 (0.9)	1
**B*39**	0 (0.0)	4 (3.5)	1
**B*40**	0 (0.0)	3 (2.7)	1
**B*41**	1 (10.0)	6 (5.3)	0.456
**B*42**	0 (0.0)	1 (0.9)	1
**B*44**	1 (10.0)	3 (2.7)	0.291
**B*47**	0 (0.0)	1 (0.9)	1
**B*49**	0 (0.0)	2 (1.8)	1
**B*50**	0 (0.0)	10 (8.8)	1
**B*51**	5 (50.0)	26 (23.0)	0.12
**B*52**	0 (0.0)	2 (1.8)	1
**B*53**	0 (0.0)	3 (2.7)	1
**B*58**	0 (0.0)	9 (8.0)	1

Abbreviations: HLA, human leukocyte antigen; COVID-19, coronavirus disease 2019.
Note: COVID Stage A: asymptomatic infection; COVID Stage B: mild infection; COVID Stage C: moderate infection; COVID Stage D: severe infection. Allele 1 and Allele 2 indicate genotyping call positions and do not imply parental origin or phased inheritance. P values were calculated using Fisher's exact test.

Supplementary Table S10. Distribution of HLA-C Allele 2 Allele Groups between COVID-19 Stage A and B+C+D

**Allele group**	**Stage A (n=10)**<br>**n (%)**	**Stage B+C+D (n=113)**<br>**n (%)**	***P* Value**
**C*01**	0 (0.0)	1 (0.9)	1
**C*02**	0 (0.0)	2 (1.8)	1
**C*03**	1 (10.0)	17 (15.0)	1
**C*04**	0 (0.0)	9 (8.0)	1
**C*05**	1 (10.0)	0 (0.0)	0.081
**C*06**	1 (10.0)	27 (23.9)	0.452
**C*07**	0 (0.0)	20 (17.7)	0.364
**C*08**	0 (0.0)	3 (2.7)	1
**C*12**	0 (0.0)	3 (2.7)	1
**C*14**	0 (0.0)	3 (2.7)	1
**C*15**	5 (50.0)	20 (17.7)	0.029
**C*16**	0 (0.0)	1 (0.9)	1
**C*17**	2 (20.0)	6 (5.3)	0.128
**C*18**	0 (0.0)	1 (0.9)	1

Abbreviations: HLA, human leukocyte antigen; COVID-19, coronavirus disease 2019.
Note: COVID Stage A: asymptomatic infection; COVID Stage B: mild infection; COVID Stage C: moderate infection; COVID Stage D: severe infection. Allele 1 and Allele 2 indicate genotyping call positions and do not imply parental origin or phased inheritance. P values were calculated using Fisher's exact test.

Supplementary Table S11. Distribution of HLA-DRB1 Allele 1 Allele Groups between COVID-19 Stage A and B+C+D

Allele group	Stage A (n=10) n (%)	Stage B+C+D (n=113) n (%)	*P* Value
**DRB1*01**	1 (10.0)	3 (2.7)	0.291
**DRB1*03**	3 (30.0)	27 (23.9)	0.705
**DRB1*04**	0 (0.0)	10 (8.8)	1
**DRB1*07**	1 (10.0)	25 (22.1)	0.687
**DRB1*09**	0 (0.0)	1 (0.9)	1
**DRB1*10**	0 (0.0)	9 (8.0)	1
**DRB1*11**	0 (0.0)	7 (6.2)	1
**DRB1*12**	0 (0.0)	2 (1.8)	1
**DRB1*13**	2 (20.0)	14 (12.4)	0.618
**DRB1*15**	3 (30.0)	13 (11.5)	0.123
**DRB1*16**	0 (0.0)	2 (1.8)	1

Abbreviations: HLA, human leukocyte antigen; COVID-19, coronavirus disease 2019.
Note: COVID Stage A: asymptomatic infection; COVID Stage B: mild infection; COVID Stage C: moderate infection; COVID Stage D: severe infection. Allele 1 and Allele 2 indicate genotyping call positions and do not imply parental origin or phased inheritance. P values were calculated using Fisher's exact test.

Supplementary Table S12. Distribution of HLA-DRB1 Allele 2 Allele Groups between COVID-19 Stage A and B+C+D

**Allele group**	**Stage A (n=10)**<br>**n (%)**	**Stage B+C+D (n=113)**<br>**n (%)**	***P* Value**
**DRB1*01**	0 (0.0)	1 (0.9)	1
**DRB1*03**	1 (10.0)	13 (11.5)	1
**DRB1*04**	2 (20.0)	30 (26.5)	1
**DRB1*07**	1 (10.0)	11 (9.7)	1
**DRB1*08**	0 (0.0)	3 (2.7)	1
**DRB1*09**	1 (10.0)	1 (0.9)	0.157
**DRB1*10**	0 (0.0)	4 (3.5)	1
**DRB1*11**	2 (20.0)	17 (15.0)	0.652
**DRB1*13**	3 (30.0)	19 (16.8)	0.383
**DRB1*14**	0 (0.0)	2 (1.8)	1
**DRB1*15**	0 (0.0)	6 (5.3)	1
**DRB1*16**	0 (0.0)	6 (5.3)	1

Abbreviations: HLA, human leukocyte antigen; COVID-19, coronavirus disease 2019.
Note: COVID Stage A: asymptomatic infection; COVID Stage B: mild infection; COVID Stage C: moderate infection; COVID Stage D: severe infection. Allele 1 and Allele 2 indicate genotyping call positions and do not imply parental origin or phased inheritance. P values were calculated using Fisher's exact test.

Supplementary Table S13. Distribution of HLA-DQB1 Allele 1 Allele Groups between COVID-19 Stage A and B+C+D

**Allele group**	**Stage A (n=10)**<br>**n (%)**	**Stage B+C+D (n=113)**<br>**n (%)**	***P* Value**
**DQB1*02**	2 (20.0)	27 (23.9)	1
**DQB1*03**	3 (30.0)	46 (40.7)	0.738
**DQB1*04**	0 (0.0)	2 (1.8)	1
**DQB1*05**	1 (10.0)	18 (15.9)	1
**DQB1*06**	4 (40.0)	20 (17.7)	0.267

Abbreviations: HLA, human leukocyte antigen; COVID-19, coronavirus disease 2019.
Note: COVID Stage A: asymptomatic infection; COVID Stage B: mild infection; COVID Stage C: moderate infection; COVID Stage D: severe infection. Allele 1 and Allele 2 indicate genotyping call positions and do not imply parental origin or phased inheritance. P values were calculated using Fisher's exact test.

Supplementary Table S14. Distribution of HLA-DQB1 Allele 2 Allele Groups between COVID-19 Stage A and B+C+D

**Allele group**	**Stage A (n=10)**<br>**n (%)**	**Stage B+C+D (n=113)**<br>**n (%)**	***P* Value**
**DQB1*02**	4 (40.0)	47 (41.6)	1
**DQB1*03**	1 (10.0)	22 (19.5)	0.686
**DQB1*05**	1 (10.0)	15 (13.3)	1
**DQB1*06**	4 (40.0)	29 (25.7)	0.455

Abbreviations: HLA, human leukocyte antigen; COVID-19, coronavirus disease 2019.
Note: COVID Stage A: asymptomatic infection; COVID Stage B: mild infection; COVID Stage C: moderate infection; COVID Stage D: severe infection. Allele 1 and Allele 2 indicate genotyping call positions and do not imply parental origin or phased inheritance. P values were calculated using Fisher's exact test.

Supplementary Table S15. Distribution of HLA-A Allele 2 Allele Groups between COVID-19 Stage A+B and C+D

Allele group	Stage A+B (n=42)<br>n (%)	Stage C+D (n=81)<br>n (%)	*P* Value
**A*01**	6 (14.3)	9 (11.0)	0.61
**A*02**	9 (21.4)	13 (16.0)	0.46
**A*03**	5 (11.9)	8 (9.9)	0.762
**A*11**	0 (0.0)	3 (3.7)	0.55
**A*23**	2 (4.8)	3 (3.7)	1
**A*24**	1 (2.4)	3 (3.7)	1
**A*26**	1 (2.4)	5 (6.2)	0.66
**A*29**	2 (4.8)	1 (1.2)	0.268
**A*30**	5 (11.9)	8 (9.9)	0.762
**A*31**	4 (9.5)	4 (4.9)	0.443
**A*32**	0 (0.0)	5 (6.2)	0.164
**A*33**	2 (4.8)	7 (8.6)	0.72
**A*34**	2 (4.8)	0 (0.0)	0.115
**A*36**	0 (0.0)	1 (1.2)	1
**A*66**	0 (0.0)	1 (1.2)	1
**A*68**	2 (4.8)	9 (11.1)	0.329
**A*74**	1 (2.4)	1 (1.2)	1

Abbreviations: HLA, human leukocyte antigen; COVID-19, coronavirus disease 2019.
Note: COVID Stage A: asymptomatic infection; COVID Stage B: mild infection; COVID Stage C: moderate infection; COVID Stage D: severe infection. Allele 1 and Allele 2 indicate genotyping call positions and do not imply parental origin or phased inheritance. P values were calculated using Fisher's exact test.

Supplementary Table S16. Distribution of HLA-B Allele 1 Allele Groups between COVID-19 Stage A+B and C+D

Allele group	Stage A+B (n=42)<br>n (%)	Stage C+D (n=81)<br>n (%)	*P* Value
**B*07**	3 (7.1)	3 (3.7)	0.41
**B*08**	5 (11.9)	14 (17.3)	0.434
**B*13**	2 (4.8)	0 (0.0)	0.115
**B*14**	0 (0.0)	2 (2.5)	0.547
**B*15**	0 (0.0)	4 (4.9)	0.298
**B*18**	1 (2.4)	1 (1.2)	1
**B*35**	3 (7.1)	5 (6.2)	1
**B*37**	1 (2.4)	1 (1.2)	1
**B*38**	0 (0.0)	1 (1.2)	1
**B*39**	1 (2.4)	3 (3.7)	1
**B*40**	0 (0.0)	3 (3.7)	0.55
**B*41**	2 (4.8)	5 (6.2)	1
**B*42**	0 (0.0)	1 (1.2)	1
**B*44**	1 (2.4)	3 (3.7)	1
**B*47**	0 (0.0)	1 (1.2)	1
**B*49**	2 (4.8)	0 (0.0)	0.115
**B*50**	3 (7.7)	7 (8.6)	1
**B*51**	15 (35.7)	16 (19.8)	0.053
**B*52**	0 (0.0)	2 (2.5)	0.547
**B*53**	0 (0.0)	3 (3.7)	0.55
**B*58**	3 (7.1)	6 (7.4)	1

Abbreviations: HLA, human leukocyte antigen; COVID-19, coronavirus disease 2019.
Note: COVID Stage A: asymptomatic infection; COVID Stage B: mild infection; COVID Stage C: moderate infection; COVID Stage D: severe infection. Allele 1 and Allele 2 indicate genotyping call positions and do not imply parental origin or phased inheritance. P values were calculated using Fisher's exact test.

Supplementary Table S17. Distribution of HLA-C Allele 1 Allele Groups between COVID-19 Stage A+B and C+D

Allele group	Stage A+B (n=42) n (%)	Stage C+D (n=81) n (%)	*P* Value
**C*01**	1 (2.4)	3 (3.7)	1
**C*02**	1 (2.4)	1 (1.2)	1
**C*03**	0 (0.0)	3 (3.7)	0.55
**C*04**	4 (9.5)	13 (16.0)	0.32
**C*05**	0 (0.0)	1 (1.2)	1
**C*06**	10 (23.8)	7 (8.6)	0.028
**C*07**	16 (38.1)	32 (39.5)	0.879
**C*08**	0 (0.0)	3 (3.7)	0.55
**C*12**	0 (0.0)	4 (4.9)	0.298
**C*14**	3 (7.1)	5 (6.2)	1
**C*15**	3 (7.1)	5 (6.2)	1
**C*16**	3 (7.1)	2 (2.5)	0.337
**C*17**	1 (2.4)	2 (2.5)	1

Abbreviations: HLA, human leukocyte antigen; COVID-19, coronavirus disease 2019.
Note: COVID Stage A: asymptomatic infection; COVID Stage B: mild infection; COVID Stage C: moderate infection; COVID Stage D: severe infection. Allele 1 and Allele 2 indicate genotyping call positions and do not imply parental origin or phased inheritance. P values were calculated using Fisher's exact test.

Supplementary Table S18. Distribution of HLA-DRB1 Allele 1 Allele Groups between COVID-19 Stage A+B and C+D

**Allele group**	**Stage A+B (n=42) n (%)**	**Stage C+D (n=81) n (%)**	***P* Value**
**DRB1*01**	2 (4.8)	2 (2.5)	0.605
**DRB1*03**	12 (28.6)	18 (22.2)	0.437
**DRB1*04**	2 (4.8)	8 (9.9)	0.492
**DRB1*07**	7 (16.7)	19 (23.5)	0.382
**DRB1*09**	0 (0.0)	1 (1.2)	1
**DRB1*10**	3 (7.1)	6 (7.4)	1
**DRB1*11**	3 (7.1)	4 (4.9)	0.689
**DRB1*12**	0 (0.0)	2 (2.5)	0.547
**DRB1*13**	7 (16.7)	9 (11.1)	0.385
**DRB1*15**	6 (14.3)	10 (12.3)	0.762
**DRB1*16**	0 (0.0)	2 (2.5)	0.547

Abbreviations: HLA, human leukocyte antigen; COVID-19, coronavirus disease 2019.
Note: COVID Stage A: asymptomatic infection; COVID Stage B: mild infection; COVID Stage C: moderate infection; COVID Stage D: severe infection. Allele 1 and Allele 2 indicate genotyping call positions and do not imply parental origin or phased inheritance. P values were calculated using Fisher's exact test.

Supplementary Table S19. Distribution of HLA-DRB1 Allele 2 Allele Groups between COVID-19 Stage A+B and C+D

**Allele group**	**Stage A+B (n=42)**<br>**n (%)**	**Stage C+D (n=81)**<br>**n (%)**	***P* Value**
**DRB1*01**	0 (0.0)	1 (1.2)	1
**DRB1*03**	3 (7.1)	11 (13.6)	0.377
**DRB1*04**	8 (19.0)	24 (29.6)	0.205
**DRB1*07**	6 (14.3)	6 (7.4)	0.336
**DRB1*08**	2 (4.8)	1 (1.2)	0.268
**DRB1*09**	2 (4.8)	0 (0.0)	0.115
**DRB1*10**	1 (2.4)	3 (3.7)	1
**DRB1*11**	7 (16.7)	12 (14.8)	0.797
**DRB1*13**	8 (19.0)	14 (17.3)	0.809
**DRB1*14**	0 (0.0)	2 (2.5)	0.547
**DRB1*15**	2 (4.8)	4 (4.9)	1
**DRB1*16**	3 (7.1)	3 (3.7)	0.41

Abbreviations: HLA, human leukocyte antigen; COVID-19, coronavirus disease 2019.
Note: COVID Stage A: asymptomatic infection; COVID Stage B: mild infection; COVID Stage C: moderate infection; COVID Stage D: severe infection. Allele 1 and Allele 2 indicate genotyping call positions and do not imply parental origin or phased inheritance. P values were calculated using Fisher's exact test.

Supplementary Table S20. Distribution of HLA-DQB1 Allele 1 Allele Groups between COVID-19 Stage A+B and C+D

**Allele group**	**Stage A+B (n=42)**<br>**n (%)**	**Stage C+D (n=81)**<br>**n (%)**	***P* Value**
**DQB1*02**	12 (28.6)	17 (21.0)	0.347
**DQB1*03**	16 (38.1)	33 (40.7)	0.776
**DQB1*04**	0 (0.0)	2 (2.5)	0.547
**DQB1*05**	6 (14.3)	13 (16.0)	0.797
**DQB1*06**	8 (19.0)	16 (19.8)	0.925

Abbreviations: HLA, human leukocyte antigen; COVID-19, coronavirus disease 2019.
Note: COVID Stage A: asymptomatic infection; COVID Stage B: mild infection; COVID Stage C: moderate infection; COVID Stage D: severe infection. Allele 1 and Allele 2 indicate genotyping call positions and do not imply parental origin or phased inheritance. P values were calculated using Fisher's exact test.

Supplementary Table S21. Distribution of HLA-DQB1 Allele 2 Allele Groups between COVID-19 Stage A+B and C+D

**Allele group**	**Stage A+B (n=42)** **n (%)**	**Stage C+D (n=81)** **n (%)**	***P* Value**
**DQB1*02**	15 (35.7)	36 (44.4)	0.351
**DQB1*03**	7 (16.7)	16 (19.8)	0.677
**DQB1*05**	7 (16.7)	9 (11.1)	0.385
**DQB1*06**	13 (31.0)	20 (24.7)	0.457

Abbreviations: HLA, human leukocyte antigen; COVID-19, coronavirus disease 2019.
Note: COVID Stage A: asymptomatic infection; COVID Stage B: mild infection; COVID Stage C: moderate infection; COVID Stage D: severe infection. Allele 1 and Allele 2 indicate genotyping call positions and do not imply parental origin or phased inheritance. P values were calculated using Fisher's exact test.

Supplementary Table S22. Distribution of HLA-A Allele 2 Allele Groups between COVID-19 Stage A+B+C and D

Allele group	Stage A+B+C (n=104) n (%)	Stage D (n=19) n (%)	*P* Value
**A*01**	11 (10.6)	4 (21.1)	0.25
**A*02**	20 (19.2)	2 (10.5)	0.521
**A*03**	10 (9.6)	3 (15.8)	0.422
**A*11**	3 (2.9)	0 (0.0)	1
**A*23**	5 (4.8)	0 (0.0)	1
**A*24**	4 (3.8)	0 (0.0)	1
**A*26**	5 (4.8)	1 (5.3)	1
**A*29**	3 (2.9)	0 (0.0)	1
**A*30**	9 (8.7)	4 (21.1)	0.12
**A*31**	8 (7.7)	0 (0.0)	0.36
**A*32**	4 (3.8)	1 (5.3)	0.57
**A*33**	8 (7.7)	1 (5.3)	1
**A*34**	2 (1.9)	0 (0.0)	1
**A*36**	1 (1.0)	0 (0.0)	1
**A*66**	1 (1.0)	0 (0.0)	1
**A*68**	8 (7.7)	3 (15.8)	0.373
**A*74**	2 (1.9)	0 (0.0)	1

Abbreviations: HLA, human leukocyte antigen; COVID-19, coronavirus disease 2019.
Note: COVID Stage A: asymptomatic infection; COVID Stage B: mild infection; COVID Stage C: moderate infection; COVID Stage D: severe infection. Allele 1 and Allele 2 indicate genotyping call positions and do not imply parental origin or phased inheritance. P values were calculated using Fisher's exact test.

Supplementary Table S23. Distribution of HLA-B Allele 2 Allele Groups between COVID-19 Stage A+B+C and D

Allele group	Stage A+B+C (n=104)<br>n (%)	Stage D (n=19)<br>n (%)	*P* Value
**B*07**	5 (4.8)	1 (5.3)	1
**B*08**	6 (5.8)	1 (5.3)	1
**B*15**	14 (13.5)	2 (10.5)	1
**B*27**	1 (1.0)	0 (0.0)	1
**B*35**	5 (4.8)	2 (10.5)	0.295
**B*37**	0 (0.0)	1 (5.3)	0.154
**B*39**	3 (2.9)	3 (15.8)	0.047
**B*40**	3 (2.9)	0 (0.0)	1
**B*41**	1 (1.0)	0 (0.0)	1
**B*42**	1 (1.0)	0 (0.0)	1
**B*44**	1 (1.0)	0 (0.0)	1
**B*47**	3 (2.9)	0 (0.0)	1
**B*49**	2 (1.9)	0 (0.0)	1
**B*50**	20 (19.2)	2 (10.5)	0.521
**B*51**	15 (14.4)	1 (5.3)	0.462
**B*53**	3 (2.9)	3 (15.8)	0.047
**B*55**	2 (1.9)	0 (0.0)	1
**B*57**	6 (5.8)	1 (5.3)	1
**B*58**	11 (10.6)	0 (0.0)	0.211
**B*73**	2 (1.9)	0 (0.0)	1

Abbreviations: HLA, human leukocyte antigen; COVID-19, coronavirus disease 2019.
Note: COVID Stage A: asymptomatic infection; COVID Stage B: mild infection; COVID Stage C: moderate infection; COVID Stage D: severe infection. Allele 1 and Allele 2 indicate genotyping call positions and do not imply parental origin or phased inheritance. P values were calculated using Fisher's exact test.

Supplementary Table S23A. Any-Call HLA-B*14 Distribution between COVID-19 Stage A+B+C and D

**Allele group**	**Stage A+B+C (n=104)**<br>**n (%)**	**Stage D (n=19)**<br>**n (%)**	***P* Value**
**B*14**	0 (0.0)	2 (10.5)	0.023

Abbreviations: HLA, human leukocyte antigen; COVID-19, coronavirus disease 2019.

Note: Any-call indicates that the allele group was counted if present in either Allele 1 or Allele 2 call position. P value was calculated using Fisher's exact test.

Supplementary Table S24. Distribution of HLA-C Allele 1 Allele Groups between COVID-19 Stage A+B+C and D

**Allele group**	**Stage A+B+C (n=104)** **n (%)**	**Stage D (n=19)** **n (%)**	***P* Value**
**C*01**	4 (3.8)	0 (0.0)	1
**C*02**	2 (1.9)	0 (0.0)	1
**C*03**	3 (2.9)	0 (0.0)	1
**C*04**	14 (13.5)	3 (15.8)	0.726
**C*05**	1 (1.0)	0 (0.0)	1
**C*06**	15 (14.4)	2 (10.5)	1
**C*07**	40 (38.5)	8 (42.1)	0.765
**C*08**	1 (1.0)	2 (10.5)	0.062
**C*12**	2 (1.9)	2 (10.5)	0.112
**C*14**	7 (6.7)	1 (5.3)	1
**C*15**	8 (7.7)	0 (0.0)	0.356
**C*16**	4 (3.8)	1 (5.3)	0.574
**C*17**	3 (2.9)	0 (0.0)	1

Abbreviations: HLA, human leukocyte antigen; COVID-19, coronavirus disease 2019.
Note: COVID Stage A: asymptomatic infection; COVID Stage B: mild infection; COVID Stage C: moderate infection; COVID Stage D: severe infection. Allele 1 and Allele 2 indicate genotyping call positions and do not imply parental origin or phased inheritance. P values were calculated using Fisher's exact test.

Supplementary Table S25. Distribution of HLA-DRB1 Allele 1 Allele Groups between COVID-19 Stage A+B+C and D

**Allele group**	**Stage A+B+C (n=104)**<br>**n (%)**	**Stage D (n=19)**<br>**n (%)**	***P* Value**
**DRB1*01**	4 (3.8)	0 (0.0)	1
**DRB1*03**	24 (23.1)	6 (31.6)	0.402
**DRB1*04**	10 (9.6)	0 (0.0)	0.358
**DRB1*07**	20 (19.2)	6 (31.6)	0.232
**DRB1*09**	1 (1.0)	0 (0.0)	1
**DRB1*10**	6 (5.8)	3 (15.8)	0.143
**DRB1*11**	7 (6.7)	0 (0.0)	0.594
**DRB1*12**	2 (1.9)	0 (0.0)	1
**DRB1*13**	14 (13.5)	2 (10.5)	1
**DRB1*15**	15 (14.4)	1 (5.3)	0.462
**DRB1*16**	1 (1.0)	1 (5.3)	0.286

Abbreviations: HLA, human leukocyte antigen; COVID-19, coronavirus disease 2019.
Note: COVID Stage A: asymptomatic infection; COVID Stage B: mild infection; COVID Stage C: moderate infection; COVID Stage D: severe infection. Allele 1 and Allele 2 indicate genotyping call positions and do not imply parental origin or phased inheritance. P values were calculated using Fisher's exact test.

Supplementary Table S26. Distribution of HLA-DRB1 Allele 2 Allele Groups between COVID-19 Stage A+B+C and D

**Allele group**	**Stage A+B+C (n=104)**<br>**n (%)**	**Stage D (n=19)**<br>**n (%)**	***P* Value**
**DRB1*01**	1 (1.0)	0 (0.0)	1
**DRB1*03**	13 (12.5)	1 (5.3)	0.694
**DRB1*04**	27 (26.0)	5 (26.3)	1
**DRB1*07**	11 (10.6)	1 (5.3)	0.69
**DRB1*08**	3 (2.9)	0 (0.0)	1
**DRB1*09**	2 (1.9)	0 (0.0)	1
**DRB1*10**	3 (2.9)	1 (5.3)	0.494
**DRB1*11**	16 (15.4)	3 (15.8)	1
**DRB1*13**	16 (15.4)	6 (31.6)	0.107
**DRB1*14**	2 (1.9)	0 (0.0)	1
**DRB1*15**	4 (3.8)	2 (10.5)	0.232
**DRB1*16**	6 (5.8)	0 (0.0)	0.589

Abbreviations: HLA, human leukocyte antigen; COVID-19, coronavirus disease 2019.
Note: COVID Stage A: asymptomatic infection; COVID Stage B: mild infection; COVID Stage C: moderate infection; COVID Stage D: severe infection. Allele 1 and Allele 2 indicate genotyping call positions and do not imply parental origin or phased inheritance. P values were calculated using Fisher's exact test.

Supplementary Table S27. Distribution of HLA-DQB1 Allele 1 Allele Groups between COVID-19 Stage A+B+C and D

**Allele group**	**Stage A+B+C (n=104)**<br>**n (%)**	**Stage D (n=19)**<br>**n (%)**	***P* Value**
**DQB1*02**	22 (21.2)	7 (36.8)	0.15
**DQB1*03**	46 (44.2)	3 (15.8)	0.022
**DQB1*04**	1 (1.0)	1 (5.3)	0.286
**DQB1*05**	16 (15.4)	3 (15.8)	1
**DQB1*06**	19 (18.3)	5 (26.3)	0.528

Abbreviations: HLA, human leukocyte antigen; COVID-19, coronavirus disease 2019.
Note: COVID Stage A: asymptomatic infection; COVID Stage B: mild infection; COVID Stage C: moderate infection; COVID Stage D: severe infection. Allele 1 and Allele 2 indicate genotyping call positions and do not imply parental origin or phased inheritance. P values were calculated using Fisher's exact test.

Supplementary Table S28. Distribution of HLA-DQB1 Allele 2 Allele Groups between COVID-19 Stage A+B+C and D

**Allele group**	**Stage A+B+C (n=104)**<br>**n (%)**	**Stage D (n=19)**<br>**n (%)**	***P* Value**
**DQB1*02**	42 (40.4)	9 (47.4)	0.57
**DQB1*03**	21 (20.2)	2 (10.5)	0.52
**DQB1*05**	14 (13.5)	2 (10.5)	1
**DQB1*06**	27 (26.0)	6 (31.6)	0.61

Abbreviations: HLA, human leukocyte antigen; COVID-19, coronavirus disease 2019.
Note: COVID Stage A: asymptomatic infection; COVID Stage B: mild infection; COVID Stage C: moderate infection; COVID Stage D: severe infection. Allele 1 and Allele 2 indicate genotyping call positions and do not imply parental origin or phased inheritance. P values were calculated using Fisher's exact test.

Supplementary Table S29. Exploratory Interaction Analysis of Age, Sex, and BMI for COVID-19 Mortality and Clinical Severity Outcomes

Outcome	Interaction tested	OR for interaction (95% CI)	*P* Value
**Death vs recovery**	Age × sex	1.17 (0.71–1.93)	0.546
**Death vs recovery**	BMI × sex	1.35 (0.67–2.71)	0.400
**Death vs recovery**	Age × BMI	0.82 (0.65–1.02)	0.077
**Stage D vs A+B+C**	Age × sex	1.18 (0.62–2.26)	0.617
**Stage D vs A+B+C**	BMI × sex	1.55 (0.73–3.25)	0.251
**Stage D vs A+B+C**	Age × BMI	1.09 (0.86–1.39)	0.462
**Stage C+D vs A+B**	Age × sex	0.60 (0.37–0.96)	0.033
**Stage C+D vs A+B**	BMI × sex	0.57 (0.31–1.06)	0.074
**Stage C+D vs A+B**	Age × BMI	1.10 (0.93–1.30)	0.271

Abbreviations: BMI, body mass index; OR, odds ratio; CI, confidence interval.
Note: Analyses were exploratory. Age was modeled per 10-year increase, BMI per 5 kg/m^2 increase, and sex as male versus female. One patient with missing BMI was excluded from the interaction models; therefore, the interaction-analysis sample size was n=122. ORs represent the interaction term in logistic regression models adjusted for the corresponding main effects.
