# Supplementary figures and images for: HLA Class I and II Variants as Potential Determinants of Clinical Severity and Mortality in Patients with COVID-19: A Prospective Study from Saudi Arabia
Source: Biomedicines. 2026 May 28;14(6):1220. doi: 10.3390/biomedicines14061220 (PMC13296798; doi:10.3390/biomedicines14061220)

# Supplementary Figure 10 - Figure 2 axis-unit detail

## Axis-unit detail

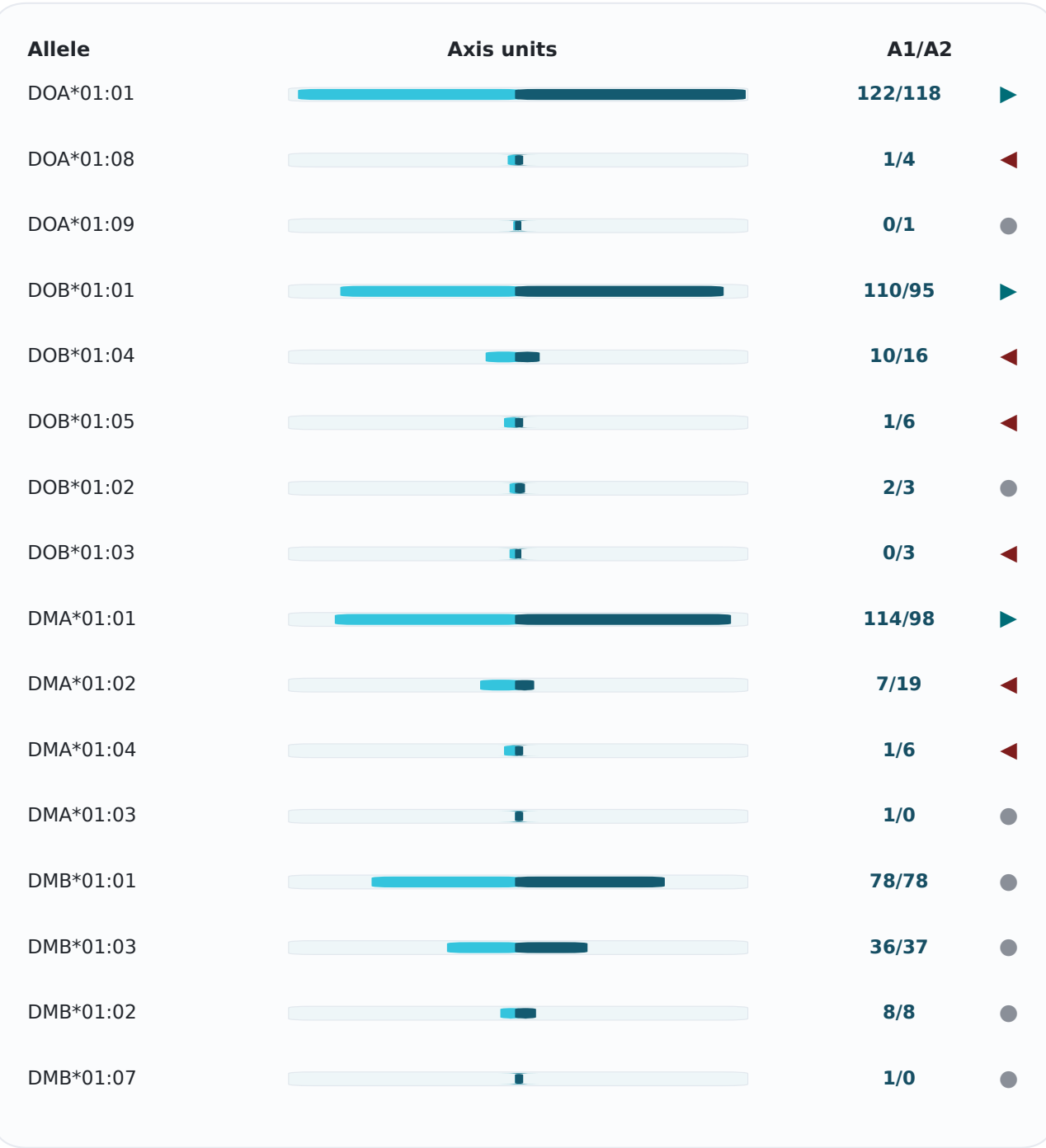

Supplement: Supplementary file 1 [file biomedicines-14-01220-s001.zip › Supplementary Figure S10.pdf]

Full paired profiles

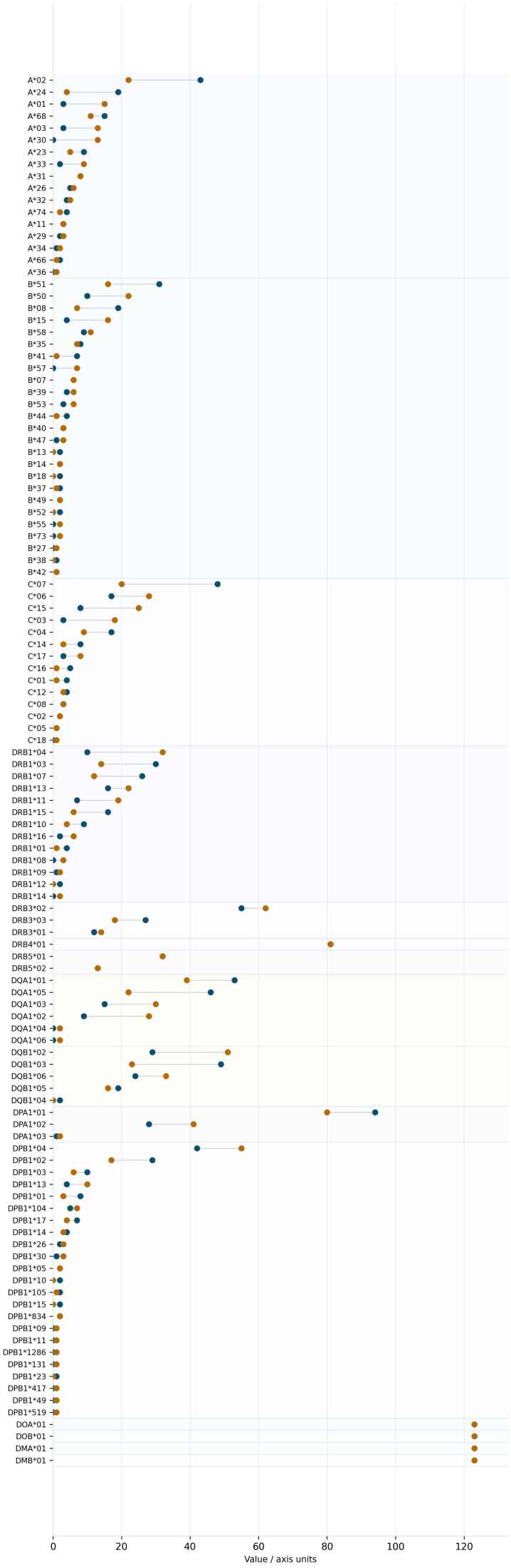

Supplement: Supplementary file 1 [file biomedicines-14-01220-s001.zip › Supplementary Figure S2.pdf]

Full dominance architecture

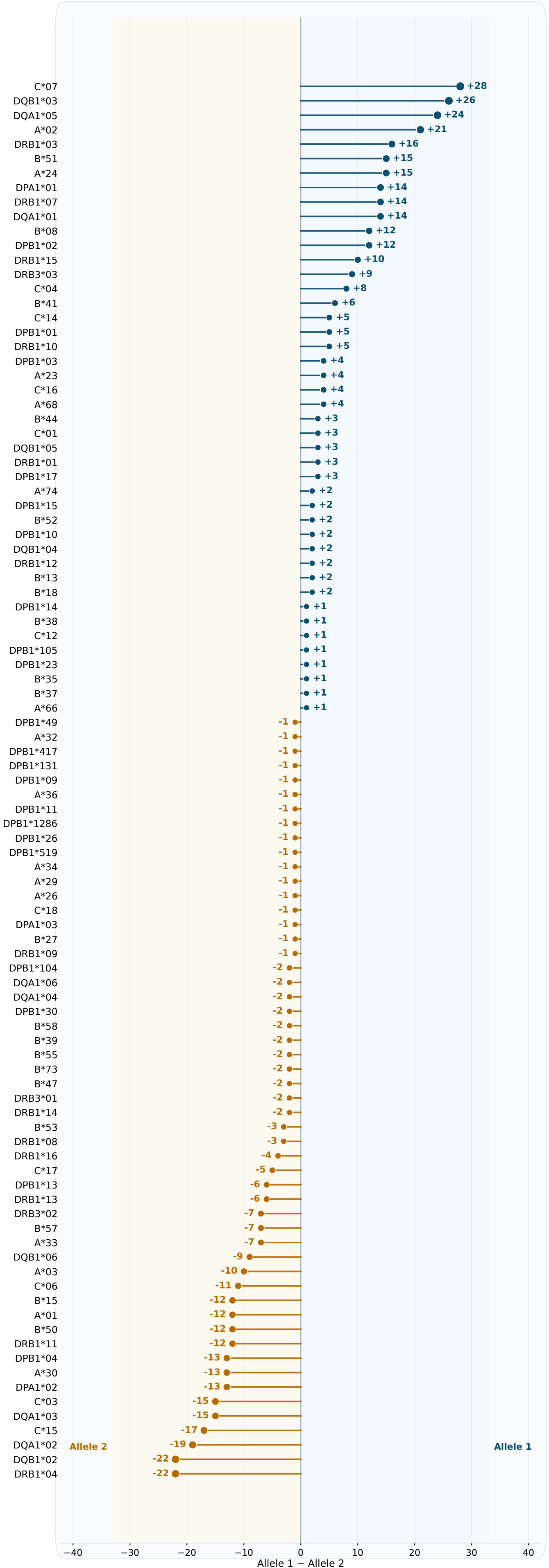

Supplement: Supplementary file 1 [file biomedicines-14-01220-s001.zip › Supplementary Figure S3.pdf]

Supplementary Figure 6 - Figure 2 full mirrored profiles

Full mirrored profiles

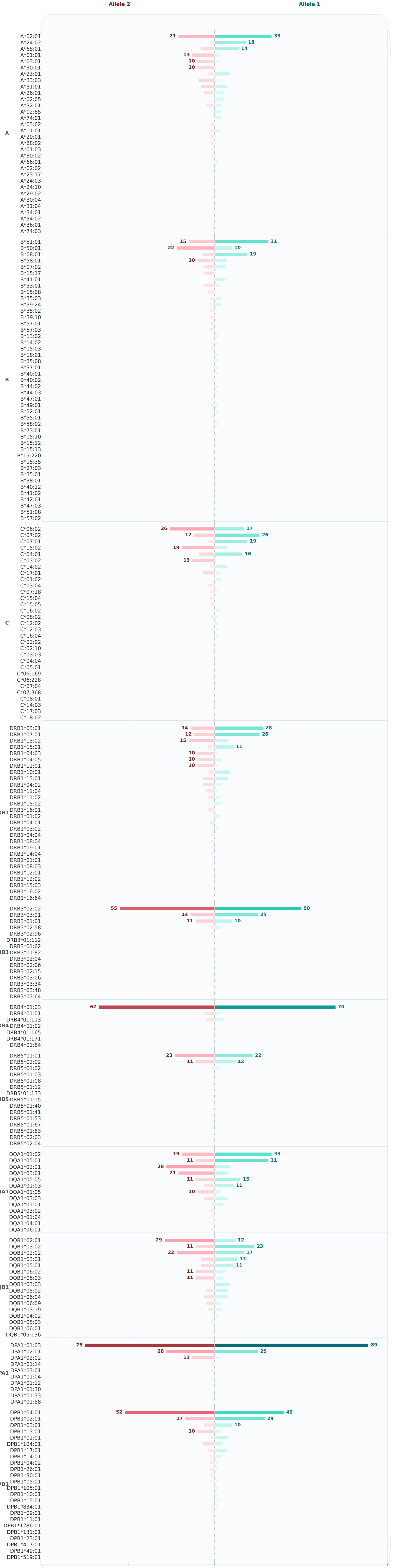

Supplement: Supplementary file 1 [file biomedicines-14-01220-s001.zip › Supplementary Figure S6.pdf]

Class I full mirrored profiles

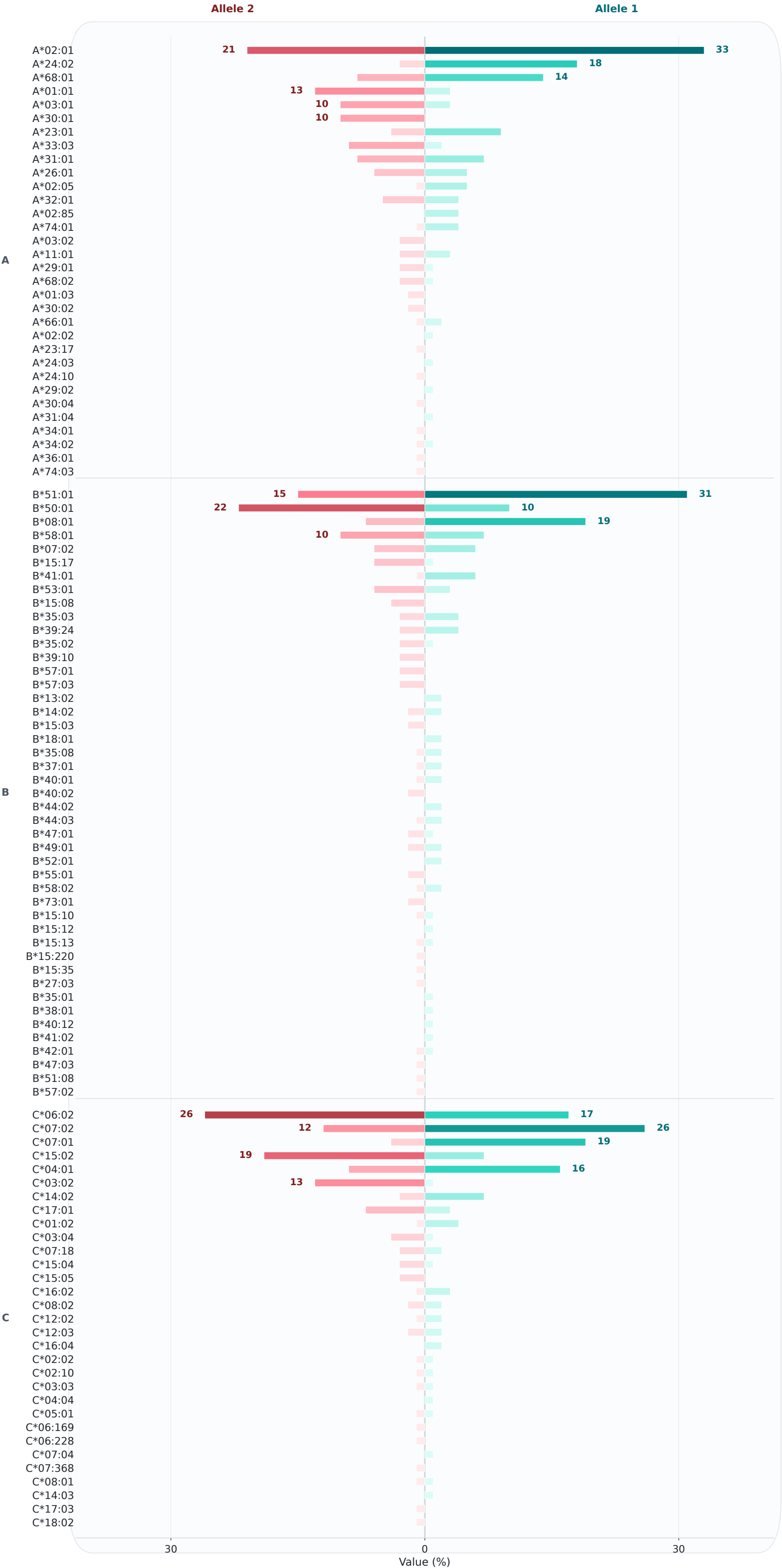

Supplement: Supplementary file 1 [file biomedicines-14-01220-s001.zip › Supplementary Figure S6A.pdf]

Supplementary Figure 6 - Figure 2 Class II full mirrored profiles

Class II full mirrored profiles

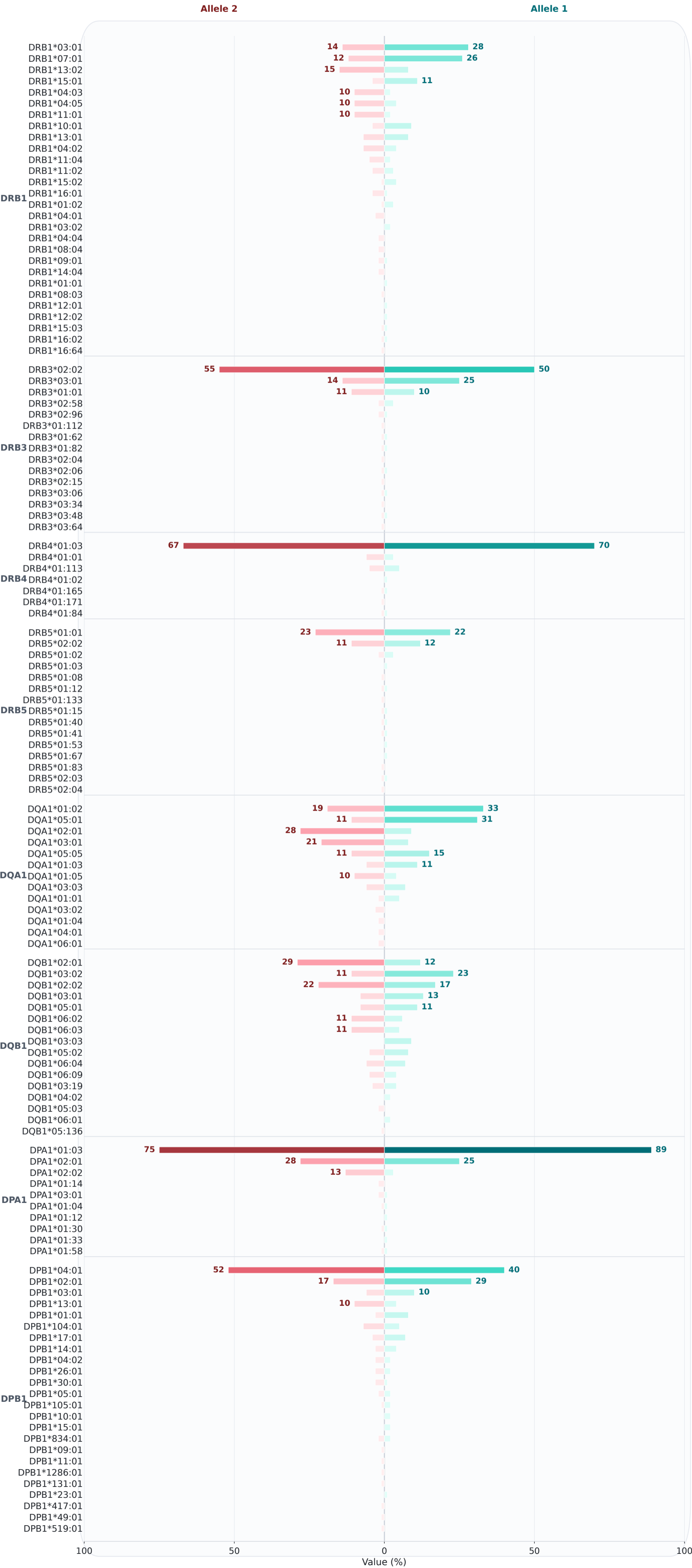

Supplement: Supplementary file 1 [file biomedicines-14-01220-s001.zip › Supplementary Figure S6B.pdf]

# Supplementary Figure 8 - Figure 2 locus diversity detail

## Locus diversity detail

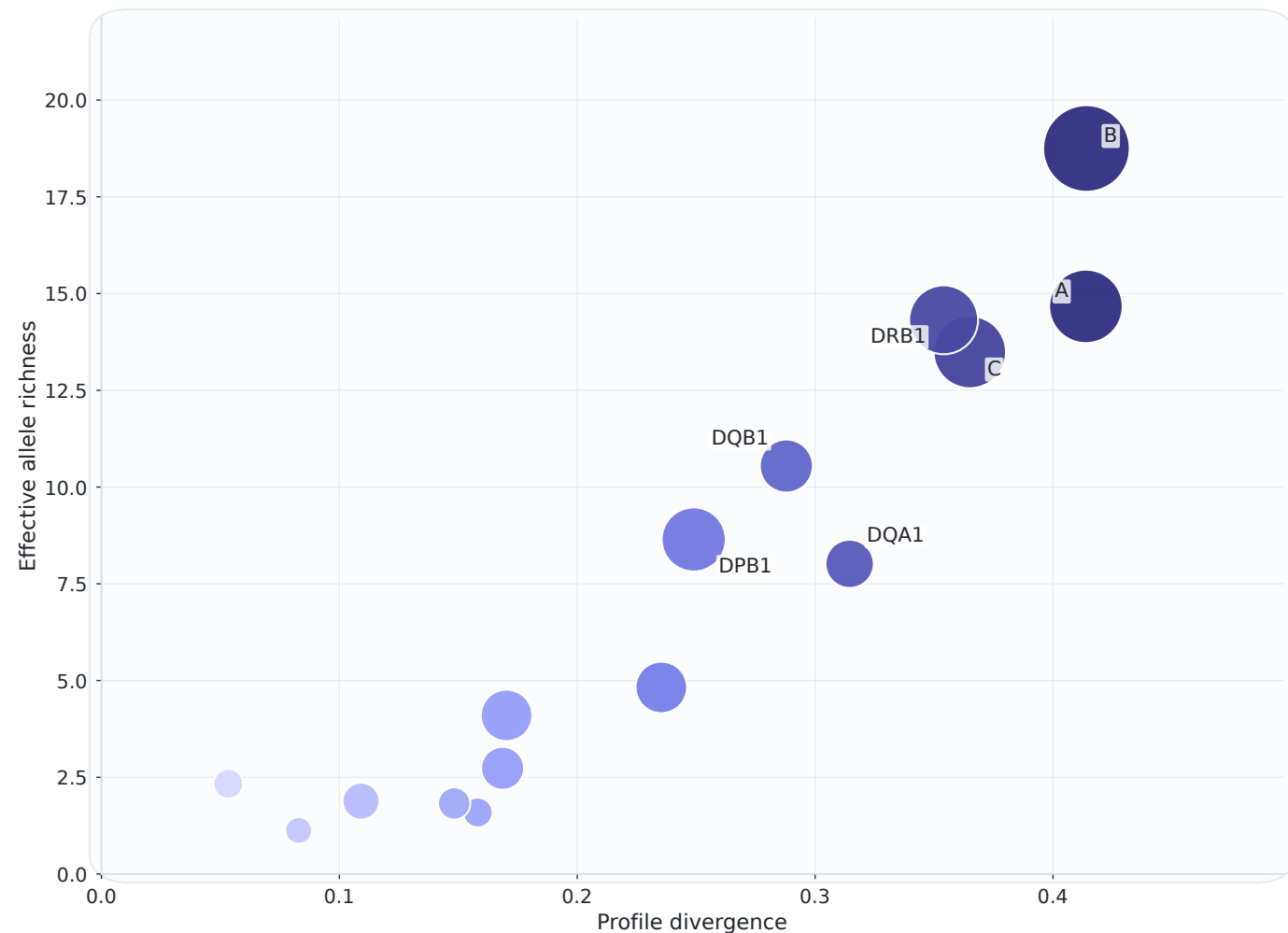

Supplement: Supplementary file 1 [file biomedicines-14-01220-s001.zip › Supplementary Figure S8.pdf]
